# The Miocene seal *Monachopsis pontica*: isolated in a shrinking sea and adapting to its changing conditions

**DOI:** 10.1098/rsos.242261

**Published:** 2025-03-19

**Authors:** Pavlo Otriazhyi, Davit Vasilyan, Karina Vishnyakova, Elena Gladilina, Pavel Gol’din

**Affiliations:** ^1^ Department of Evolutionary Morphology, Schmalhausen Institute of Zoology, National Academy of Sciences of Ukraine, Kyiv, Ukraine; ^2^ Department of Geosciences, University of Fribourg, Chemin du musée 6, Fribourg 1700, Switzerland; ^3^ JURASSICA Museum, Route de Fontenais 21, Porrentruy 2900, Switzerland; ^4^ Ukrainian Scientific Centre of Ecology of the Sea, Odesa, Ukraine; ^5^ BioEcoLinks NGO, Lymanka, Odesa, Ukraine

**Keywords:** pinnipeds, Phocidae, Paratethys, Miocene, skeleton, phylogeny

## Abstract

More than 170 years have passed since the description of the dwarf Miocene seal *Monachopsis pontica*. However, its cranial materials were rarely found and described. This paper re-describes *M. pontica* and discusses its ecological adaptations. *M. pontica* is among the latest seals that survived in the epicontinental sea Eastern Paratethys during the Khersonian biotic crisis. Newly examined materials from Ukraine, being exceptional in their completeness, show previously unknown traits of its morphology, unique among subfamily Phocinae. *M. pontica* is distinguished by its small body size (85–105 cm long), long snout and long deltoid crest of the humerus. Dental morphology shows that *M. pontica* was using raptorial methods of catching prey. However, the pattern of tooth wear also indicates suction feeding. The unusually small body size could be interpreted as a result of the decreasing size of the basin and the absence of predators. High crests on limb bones show evidence of the development of musculature driven by pachyosclerosis of the skeleton. Phylogenetic analysis placed *M. pontica* at the base of the crown Phocinae, crownward to the most known Miocene seals.

## 1. Introduction

The Paratethys was a large epicontinental sea in Eurasia that existed during the Oligocene and Miocene [1–4]. Between the latest Middle Miocene and the earliest Late Miocene, the Paratethys formed an isolated basin, which subsequently fell apart into the Central and Eastern Paratethys [5–8]. This age is often referred to as the Sarmatian *sensu lato* which is divided into the Volhynian, Bessarabian and Khersonian substages. Geographical isolation made the Eastern Paratethys a place of evolution and endemism of many marine life forms in Volhynian and Bessarabian (e.g. fishes [9] and marine mammals [10–12]). However, in the late Bessarabian and during the Khersonian substage, the Eastern Paratethys passed through a few drying periods and salinity crises [13–15]. These changes led to the decline of endemic fauna of fishes (e.g. [16]) and marine mammals [11,12,17].

True seals (Phocidae) were among the aquatic animals which were highly speciated during the late Middle and early Late Miocene in the Eastern Paratethys [11]. At least a dozen seal species have been described from the Bessarabian age of the Eastern Paratethys [11,18]. Most of the seal findings were incomplete postcranial skeletal elements like ribs, humeri or femora, and their taxonomic description was mostly based on those elements; also, lots of type materials are fragmentary, and some of them include only isolated or even incomplete humeri or femora [11,19,20]. On the other hand, cranial materials were rarely found that precluded firm conclusions on the taxonomy, evolution and phylogeny of Paratethyan seals. As a result, recent studies have suggested putting most, if not all, Paratethyan taxa as *nomina dubia* and re-started their taxonomic revision from scratch [21].

*Monachopsis pontica* [22] was the first seal species described from the Paratethyan region. Eichwald [22] originally described this species as *Phoca pontica* based on the cranial (including nasal bones and teeth) and postcranial elements (vertebral column, limb bones) from Ak Burun Cape, Kerch Peninsula, Ukraine. The association of the bones with each other was unclear but since these bones shared the same distinctive morphological traits, were of the same size category, shared similar preservation features, originated from the same locality and age, and no other taxa have been reported from that locality and horizon since 1850, we considered them as those which belong to the same species. Other authors also added the description of this species based on new findings. Alekseev [23] associated a newly found rostral part with the cranium described by Eichwald [22]. Malik & Nafiz [24] made a description of the Miocene fauna of Küçükçekmece, Turkey, in which they mentioned the cranial material of *Ph. pontica*. Kretzoi [25] erected the new genus of *Monachopsis*. Kirpichnikov (1964) compared the composite skeleton of *M. pontica* with *Pusa caspica* but this publication did not include illustrations or collection numbers. Koretsky [26,27] described sexual dimorphism in the postcranial bones of *M. pontica* and showed new morphological traits of this species based on the newly found maxilla. Also, Koretsky [11] reviewed the history of the studies of *M. pontica* and summed up its previous descriptions, reviewed the original type series, and reported that only one left humerus remained, so this bone was chosen as the neotype (see §2). However, Saburov [28] found a hitherto hidden part of the type series and published photos of the humerus and femur, both previously illustrated in the original publication by Eichwald [22]. Also, one of the authors of this paper (P.G.) identified other elements of the type series including the cranial fragments, as reported in §2. Therefore, *M. pontica* is among those Paratethyan seal species whose original description was based not only on isolated postcranial bones but also on cranial material. Overall, as can be concluded from the original description and illustrations by Eichwald [22] and the published research of the type series, *M. pontica* can be distinguished in its small body size, one of the smallest among all true seals, long and narrow nasal bones, and short and thick humerus and femur bones. *M. pontica* lived in the last period of the existence of the Sarmatian Sea and possibly survived during the ecosystem degradation and biotic changes caused by the Khersonian crisis.

The original description and later studies only briefly mentioned cranial materials. Therefore, new and more complete specimens from other Khersonian localities of the Kerch Peninsula allow us to revise old specimens and update the genus description with the new data and illustrations of the dentition and rostral morphology. The new cranial material also helps us to propose hypotheses about the ecology of *M. pontica* and its position within the pinniped phylogenetic tree. A review of the previous findings and a description of the new ones allow us to show the geographical distribution of *M. pontica* and draw suggestions about its ecology under crisis conditions of Paratethys shrinking, salinity changes and ecosystem degradation.

### 1.1. Material and methods

#### 1.1.1. Institutional abbreviations

FFE, Feldman Family Museum, Kharkiv, Ukraine; GNM, Georgian National Museum, Tbilisi, Sakartvelo (Georgia); IZ NASU, Schmalhausen Institute of Zoology, National Academy of Sciences of Ukraine, Kyiv, Ukraine; KNU, Taras Shevchenko Kyiv National University, Kyiv, Ukraine; LPB(UFBG), Bucharest University, Romania; MAB, Oertijdmuseum de Groene Poort, Boxtel, The Netherlands; MNEIN, National Museum of Ethnography and Natural History, Chișinău, Moldova; MPGl, Gornyi Institute (Berginstitut), St Petersburg, Russian Federation; NMB, Natural History Museum Basel, Basel, Switzerland; NMBE, Natural History Museum of Bern, Bern, Switzerland; NMNH, Muséum national d’Histoire naturelle, Paris, France; NMNHU-P (before 1998 known as IZUAN), National Museum of Natural History at the National Academy of Sciences of Ukraine, Kyiv, Ukraine; NWM, Natural History Museum Vienna, Vienna, Austria; ONU, Mechnikov Odesa National University, Odesa, Ukraine; SNM Slovak National Museum, Bratislava, Slovakia; TNU, V.I. Vernadsky Taurida National University, Simferopol, Ukraine; USNM, Department of Paleobiology, National Museum of Natural History, Washington, DC, USA; ZMUC, Natural History Museum of Denmark, Copenhagen, Denmark.

#### 1.1.2. Material

This study is based on the type series (MPGl 113), previously described specimens [11,28], undescribed specimens from several localities in Crimea and several more recently found specimens, which were collected on the Khroni Cape, Kerch Peninsula (Crimea, Ukraine; approximate geographical coordinates 45.4392, 36.5774), in 2010−2012.

Previously described materials include the cranium MPGI NA-113, the left humerus MPGl 16-113, the femur MPGl 25-113 [22], other bones from the type series and associated materials from the MPGl collections [28] from Kerch (Crimea, Ukraine) (partially examined by P.G. in 2010) and specimens from the collection NMNHU-P described by [11] (maxilla NMNHU-P 64-516, humeri (NMNHU-P 64-170) from Uzunlar and femora (NMNHU-P 64-405, 64-412) from Kamysh-Burun, femora (NMNHU-P 64-314) from Kyz-Aul (all from Crimea, Ukraine)).

New referred material is listed in table 1.

**Table 1.** The list of newly described materials.

collection and number	bone elements	location	other information
NMNHU-P	radii (NMNHU-P 64-370), femora (NMNHU-P 64-461)	Kerch, Kerch peninsula, Crimea, Ukraine.	
NMNHU-P	humeri (NMNHU-P 64-257) femora (NMNHU-P 64-254, 64-256, 64-357, 64-361, 64-362)	Kamysh-Burun, Kerch peninsula, Crimea, Ukraine (45.283, 36.400)	
NMNHU-P	humeri (NMNHU-P 64-248)	Kyz-Aul, Kerch peninsula, Crimea, Ukraine (45.083, 36.350)	
NMNHU-P	humeri (NMNHU-P 64-258, 64-714), radii (NMNHU-P 64-260), femora (NMNHU-P 64-453, 64-454, 64-456 64-457), astragali (64-348)	Kerch peninsula, Crimea, Ukraine	
NMNHU-P	humeri (NMNHU-P 64-171)	Uzunlar, Kerch peninsula, Crimea, Ukraine (45.075, 36.107)	
TNU CH00	TNU CH00-01 rostral part of the skull, CH00-02 sacrum, a cervical vertebra (possibly C5) CH4-13, and a femur CH4-17	Khroni Cape, Kerch peninsula, Crimea, Ukraine (45.439, 36.581)	TNU CH00-1 includes a proximal part of the skull with well-preserved nasal bones and tooth alveoli (figure 1). The specimen was collected by K.V. in 2010. The IZ NASU temporarily houses the specimen
FFM 10246	skull, mandible, vertebral column, ribs, right scapula, humeri, radiuses, left ulna, most of the left carpus, left metacarpus and left forelimb phalanges, ilium, femurs, tibias, fibulas, and most of the hind flippers belong to one individual left scapula, right ulna, part of left fore flipper, and right calcaneus belong to other individuals other bones are reconstructed (see figure 2)	Khroni Cape, Kerch peninsula, Crimea, Ukraine (45.439, 36.581)	collected in 2011. The skeleton is a composite of at least two individuals and includes some reconstructed elements (more details in figure 2). The fossils originated from the same locality and layers
TNU CH05	1. mandible, isolated teeth, a sternum, a scapula, a humerus, left tibiae and fibula, proximal part of right tibiae, tarsal, and multiple phalanges. 2. radius, calcaneus, cuboid, tibia and fibula, three caudal vertebrae, multiple rib fragments, and multiple phalanges. 3. juvenile tibia and few phalanges	Khroni Cape, Kerch peninsula, Crimea, Ukraine (45.439, 36.581)	the collection TNU CH05 includes three individuals (marked by numbers). P.G. and E.G. collected them in 2012. Currently temporarily housed by the IZ NASU
TNU T007	two femora	Lake Töbeçik, Kerch peninsula, Crimea, Ukraine (45.153, 36.398)	collected on the outcrops near the southeastern shore of Lake Töbeçik by P.G. in 2010. The original materials were not available for this research and were described based on photos taken by P.G. in 2012
TNU T010	four humeri and two femora	Lake Töbeçik, Kerch peninsula, Crimea, Ukraine (45.168, 36.333)	collected on the outcrops south of Lake Töbeçik by P.G. in 2010. The original materials were not available for this research and were described based on photos taken by P.G. in 2012
collection PivdenNIRO	five heads of scapula, five humeri, eight radii, two proximal parts of ulnae, eight coxae, and eight femora	Khroni Cape, Kerch peninsula, Crimea, Ukraine (45.439, 36.581)	collected by Sergei Rebik and Valentin Serbin in 2006−2009. Bones are isolated and do not have any collection numbers. The original materials were not available for this research and were described based on photos taken by P.G. in 2012
ONU	radius, a rib, a sacrum, and a pelvis	Kyz-Aul, Kerch peninsula, Crimea, Ukraine (45.083, 36.350)	bones are without collection numbers and were collected by Mykhailo Sydorenko *ca* 1910

**Figure 1.**
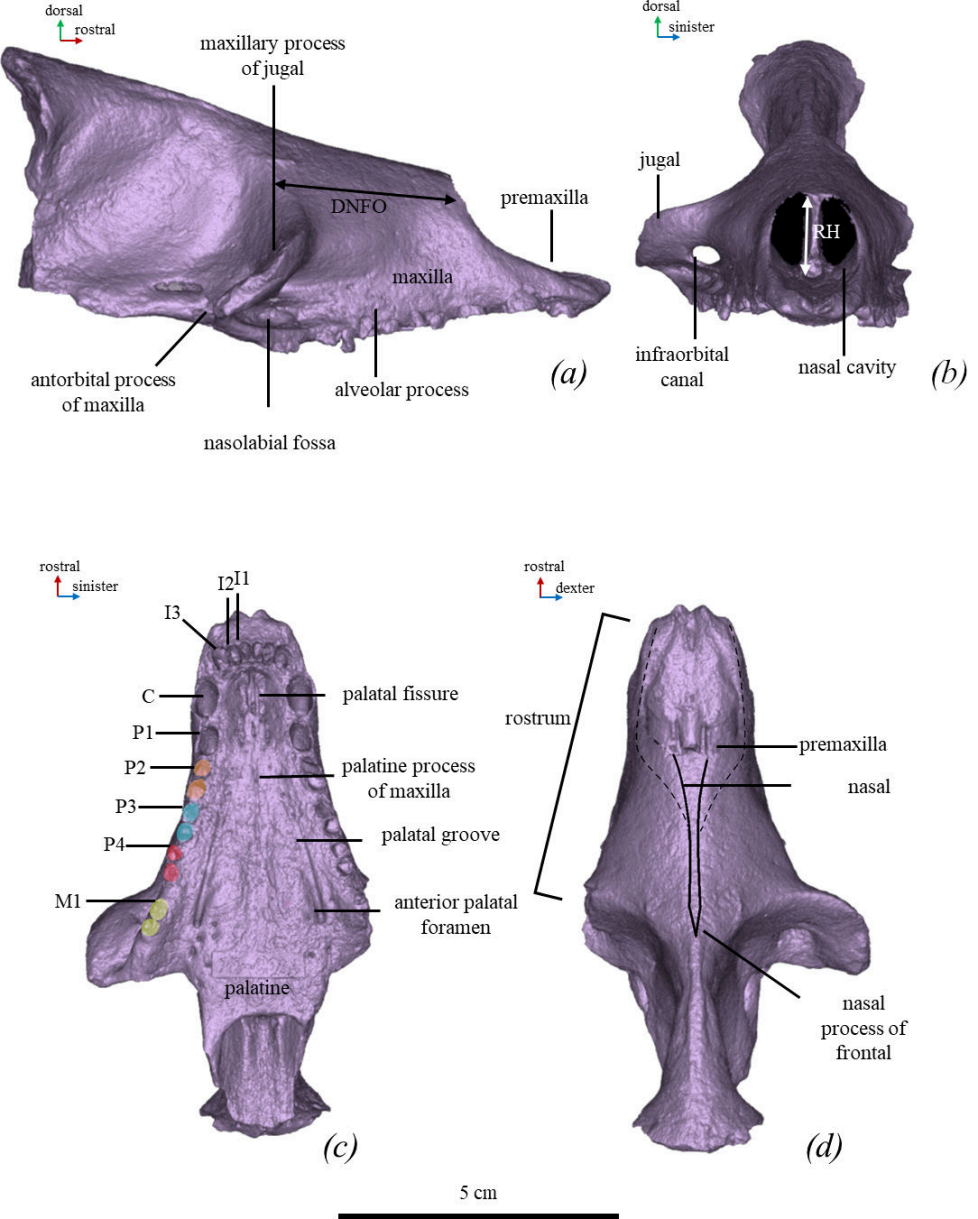
Skull of *M. pontica* (TNU CH00-01). Digital models of the bones. In (*a*) lateral, (*b*) anterior, (*c*) ventral and (*d*) dorsal views.

**Figure 2.**
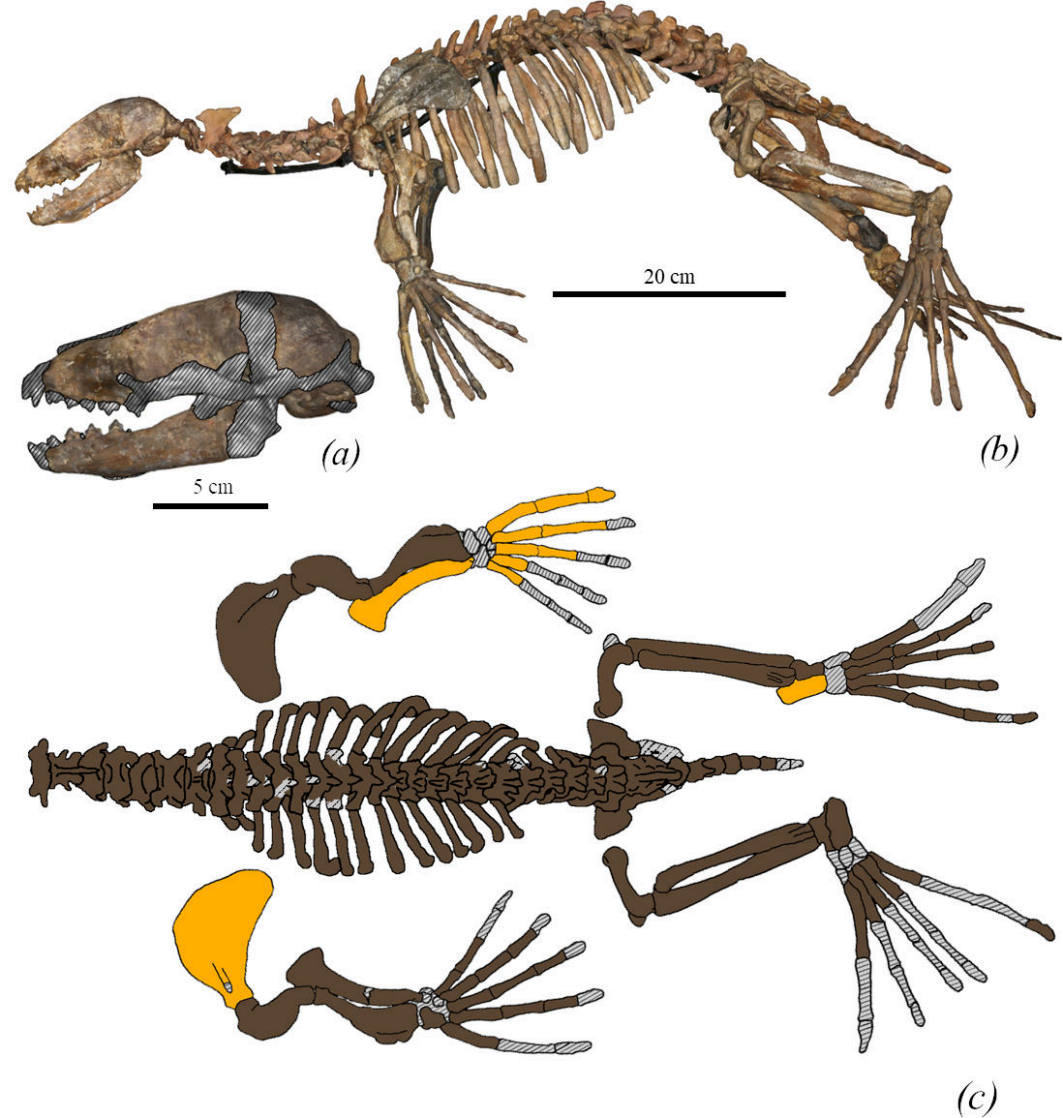
Three-dimensional models of the skeleton of *M. pontica* based on the surface scan of FFM 10246. (*a*) The skull. Grey hatching—reconstruction. (*b*) General view of the skeleton of FFM 10246. (*c*) Postcranial bones of FFM 10246. Brown colour demonstrates fossils from one individual, orange—from other individuals and hatching—reconstruction.

The list of comparative materials is provided in electronic supplementary material, table S1.

#### 1.1.3. Methods

For comparison, three-dimensional surface scans of the bone material have been obtained using the three-dimensional surface scanner Artec Space Spider. The three-dimensional models have been processed by Artec Studio 17 software under the Global registration and Sharp fusion algorithms. Fossils and bones of extant species of true seals were scanned for this study in collections from Austria, Denmark, Hungary, Moldova, Romania, Sakartvelo (Georgia), Slovakia, Switzerland and Ukraine (the list of the collections and specimens is provided in electronic supplementary material, table S1). Also, we used photos of modern seal bones from the Idaho Virtual Museum (virtual.imnh.iri.isu.edu).

For measurements, a scheme by Ericson & Storå [29] was used. Measurements were taken using electronic callipers (see electronic supplementary material, tables S2–S4), Fiji software [30] was used for the pictures of bones from the Idaho Virtual Museum and a measuring tool in Artec Studio software was used for three-dimensional models (see electronic supplementary material, tables S2 and S3). For mandibular and dental measurements, we used a modified scheme from Valenzuela-Toro *et al.* [31].

The body length estimates were based on power regression of humerus length on snout-to-end-of-tail body length. For the regression analysis, five species were used: *Erignathus barbatus*, *Cystophora cristata*, *Histriophoca fasciata*, *Pusa hispida* and *Phoca vitulina* (see electronic supplementary material, table S4, for details). The regression was calculated based on the body length and the humeral greatest length. A juvenile *C. cristata* was added to the dataset to show a specimen similar in size to *M. pontica*. In addition, a mounted and reconstructed skeleton, FFM 10246, was measured. For comparison, the body size of other dwarf Miocene seals was also estimated, among them, *Australophoca changorum*, ‘*Batavipusa neerlandica*’ MAB 3789, *Nanpophoca vitulinoides*, ‘*Praepusa boeska’* MAB 4686 and *Praepusa procaspica* (see electronic supplementary material, table S4 and references therein).

The anatomical nomenclature was based on publications on the anatomy of Miocene seals [11,32]. Also, the anatomy of domestic dogs has been used [33–35].

The character–taxon matrices for the phylogenetic analysis by Dewaele *et al*. [32] have been adopted. We excluded species with less than 15 available morphological characters (*P. boeska*, *P. pannonica*). We coded and added *M. pontica* (for most bones we used FFM 10246 and for the nasal, and premaxilla TNU CH00-01, respectively) to the matrix. In total, the matrix included 33 taxa. For the morphological matrix, we implemented the combination of traits and species from matrices from Dewaele *et al*. [32]. Also, two characters (89 and 90) were added from Koretsky [11]. In total, it included 90 morphological characters (see electronic supplementary material). For calibration of the tree, molecular sequences of modern taxa were included. We took four genes from [36] (FLVCR1, PNOC, RAG1, RAG2) and the Cytochrome b gene [37] (see electronic supplementary material, table S5).

The phylogenetic analysis was made using two approaches: maximum parsimony and total evidence with fossilized birth–death analysis. Total evidence [38] with fossilised birth–death [39] analysis was made with BEAST 2.5 [40] using packages sampled-ancestor 1.1.3 and morph-models 1.0.2. The site model for molecular data was four categories for the Gamma variation model and estimate *Shape* parameter. For morphological data, we used Lewis Mk with no Gamma variation. We applied the Relaxed Clock Log Normal model [41], and the fossilized birth–death model as tree prior distribution with a starting value of diversification rate of 0.05, and Rho was estimated. The chain length was set on 15 million generations. The log was made every 1000 trees. Two runs of the analysis were made. The convergence of two runs was checked visually in Tracer [42] for all parameters. The trees were summarized in TreeAnnotator 2.7.7. Maximum parsimony was performed using TNT 1.6 software [43]. For the phylogeny of modern taxa, we used a constrained molecular tree from Fulton & Strobeck [36] with the addition of *Otaria flavescens* as an outgroup. In parsimony analysis, we used only morphological data for both modern and fossil taxa. Wagner tree setting was used with 10 000 iterations, with 10 trees saved from each, and implied weighting (*k* = 3) was turned on. Tree bisection reconnection was used as a swapping algorithm; also, the replace existing trees option was turned on. Results were outputted as frequency differences—GC. The consistency index and retention index were obtained with STATS.RUN module.

### 1.2. Geological setting

The Serravallian and Tortonian ages in the Paratethys region are described in terms of regional stages related to changing connectivity to the global ocean, isolation of the basins, development of the Sarmatian sea and evolution of endemic Paratethyan faunas in the highly dynamic environment [44,45]. The Sarmatian *sensu lato* deposits in the Eastern Paratethys are divided into Volhynian (12.65–12.05 Ma), Bessarabian (12.05–9.9 Ma) and Khersonian (9.9–7.65 Ma [46]) regional substages [45,47,48]. The next stage after Khersonian in the Eastern Paratethys is the Maeotian stage (7.65–6.1 Ma) [45,49]

**Figure 3.**
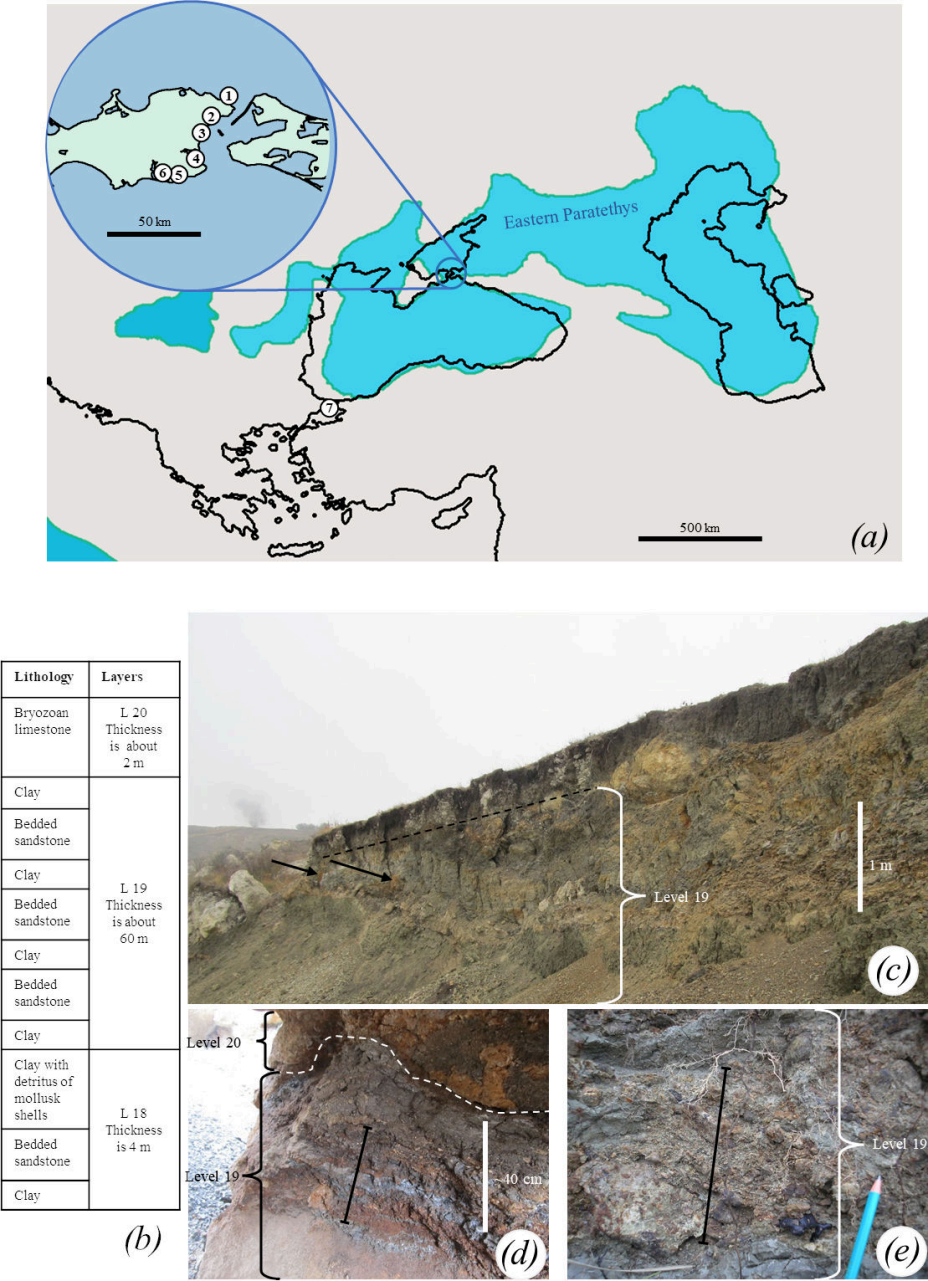
Localities with *M. pontica* findings. (*a*) Reconstruction of Eastern Paratethys shoreline during the Khersonian [51] with modern outlines of European seas. (*b*) The stratigraphic column of the Jurkine section (part of the Khroni cape) with lithology, adapted from [9], is not scaled. (*c–e*) The layers at the Khroni Cape are where the examined specimens of *M. pontica* come from. (*c*) Arrows point at laminated dark grey clays. (*d*) The bar marked clays with red terrigenic sediments. (*e*) The bar marked flaggy-bedded sandstone. Localities with *M. pontica* record: 1—Khroni Cape (Crimea, Ukraine). 2—Ak Burun and Mt Mithridat (Kerch, Crimea, Ukraine). 3—Kamysh-Burun (Kerch, Crimea, Ukraine). 4—Töbeçik (Crimea, Ukraine). 5—Kyz-Aul (Qız-Aul) (Crimea, Ukraine). 6—Uzunlar (Crimea, Ukraine). 7— Küçükçekmece (Istanbul, Turkey).

In the original description by Eichwald [22], two main locations with *Phoca pontica* findings were mentioned, with a distance of 3.5 km between them. According to Eichwald [22], the layers with seal fossils are described as layers with ferruginous clay with selenite crystals, included between layers of clays with shale structure and marl. The first locality is the top layers of the Ak Burun Cape, Kerch Peninsula, Ukraine, where the type series was found. The stratigraphy of this location is only verbally described in the literature [13,50], without any log, and well-constrained ages. The second locality is Mithridat Mountain in Kerch, Ukraine. Its stratigraphy was described by Andrusov [13]. The fossiliferous layers, with similar lithologies as described by Eichwald [22], are indicated as horizons 3, 4 and 5. The layer 3 consisted of light grey clays and two layers of marls with *Chersonimactra caspia* casts. The layer 4 consisted of marls laying over light grey clays with ferruginous clays with selenite crystals under them. The layer 5 consisted of marls with *Chersonimactra caspia* casts. Therefore, based on the presence of *Chersonimactra caspia*, these layers can be correlated to the Khersonian substage. The exposed sequence at Mithridat Mountain was destroyed by construction works several decades ago and is currently unavailable for reexamination (see these and other localities in figure 3). The sequence at the Khroni Cape where the newly examined specimens were found is represented by Bessarabian, Khersonian and Maeotian deposits and was recently described by [9]. The seal bones were found in the uppermost Khersonian horizon (level 19 *sensu* [9]) covered by clays with breccia or redeposited in the Maeotian horizon in a terrigenic layer between limestone reef blocks (figure 3). The level 19 contains shales of *Chersonimactra caspia* and is thick—up to 60 m. It consists of dark grey laminated clays with flaggy-bedded sandstone interbeds (up to 10 cm). The level 19 lays on greenish-grey clays with intercalations of sandstone and debris of *Chersonimactra caspia* (the upper part of level 18, the total thickness of the level is 4 m) and overlain by the Maeotian bryalgal limestone (level 20 [9]).

Also, fossils from other localities originated from the Bessarabian–Khersonian boundary (Töbeçik Lake) to the Khersonian layers (see Other localities and ages).

## 2. Systematic palaeontology

Family Phocidae Gray, 1821

Subfamily Phocinae Gray, 1821

Genus *Monachopsis* Kretzoi, 1941

Type (and only) species *Monachopsis pontica* Eichwald, 1850

The original species name and its synonyms:

*Phoca pontica* Eichwald, 1850

*Phoca pontica* Alekseev 1924

*Phoca pontica* Malik and Nafiz 1933

*Monachopsis pontica* [25]

*Monachopsis pontica* [52]

*Phoca pontica* [53]

*Phoca pontica* [54]

*Phoca pontica* [10]

*Monachopsis pontica* Koretsky, 1987, 1988, 2001 [55,56]

*Monachopsis pontica* [28]

**Figure 4.**
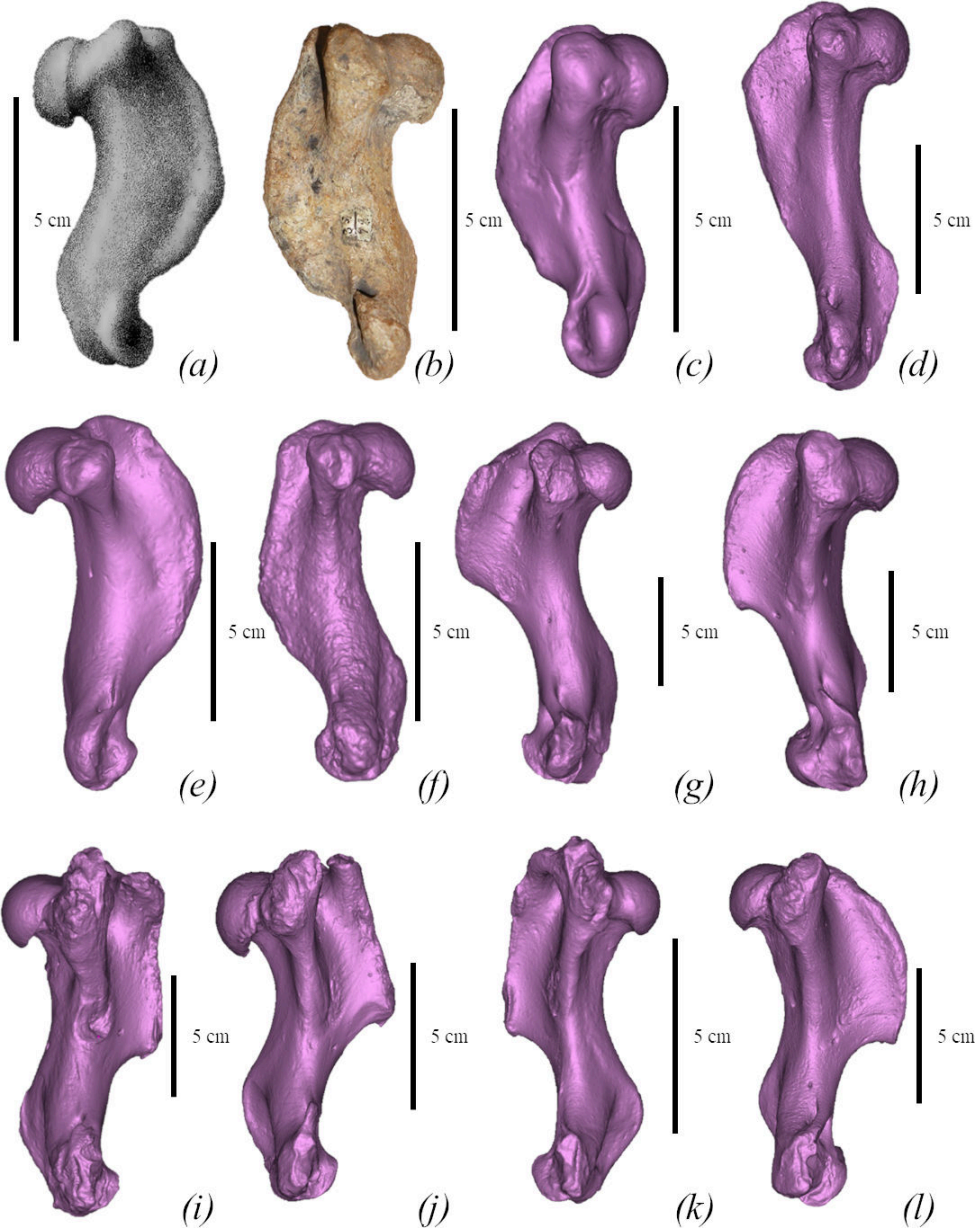
Comparison of humeri of true seals (Phocidae) in medial view. (*a*) *M. pontica* MPGl 17-113, left. (*b*) *M. pontica* MPGl 48-113, right. (*c*) *M. pontica* NMNHU-P 64-257, right. (*d*) *D. emryi* SNM Z 25507, right. (*e*) *Cr. maeotica* NMNHU-P 64-530, left. (*f*) *Cr. maeotica* NMNHU-P Nordman, right. (*g*) *E. barbatus* CN 958 (NHMD), right. (*h*) *C. cristata* 1134 (NHMD), right. (*i*) *Ha. grypus* 1485 (NHMD), left. (*j*) *Ph. vitulina* NMBE 301-91, left. (*k*) *Pu. caspica* NMW 66298, right. (*l*) *Pa. groenlandicus* CN 961 (NHMD), left.

*Type specimen.* The type series (MPGl 113) originally included a neurocranium fragment with a left tympanic bone, a canine, postcanine teeth, a partial scapula, humeri, an ulna, a radius, a fragment of the sacrum, coxae, femora, a partial tibia, vertebrae, a sacrum, an astragal, a calcaneus, a cuboid, metatarsals and a phalanx (see original illustration in electronic supplementary material, figure S1). Also, the original description included nasal bones, which were not illustrated. Eichwald [22] described the type series without mentioning if bones were associated and did not erect a holotype. In 1960, McLaren [52] chose the cranium as the lectotype. However, Koretsky [11] suggested that the cranium was supposed to be destroyed during the Second World War, and most other types of materials were supposedly lost. Therefore, the left humerus (MPGl 17-113) was designated as the neotype by Koretsky [11] based on the following: it belonged to the type series and the type locality, and it was described and illustrated by [22,57, plate 13, no. 17] (see electronic supplementary material, figure S1). Meanwhile, Saburov [28] reported several bones from the type series found in the MGPI collections. Also, one of the authors (P.G.) personally examined the type series in 2010 and found that it still included the following bones: a cranial fragment including a partial basicranium and a tympanic bulla, a rostral fragment, vertebrae, a humerus MPGl 48-113 (figure 4), fragments of humeri, radius, fragments of ulna, fragments of femora and fragments of tibia. Therefore, we can conclude that the type series is mostly preserved, consistent with the illustrated and commented original description and it can be used for taxonomic study and nomenclatural use. As no other taxa were found or identified from that series or locality, we are assuming that the whole type series belongs to the same species, but it is still unclear which part of the type series belongs to the same individual. Also, the partial cranium (MPGI NA-113), although mostly destroyed and only suggested as reconstructed from the illustration and original description, is formally retained as the lectotype. Therefore, we also consider *Monachopsis pontica* as a valid name.

*Type locality and age.* Ak Burun Cape, Kerch, Crimea, Ukraine (geographical coordinates 45.318, 36.493), outcrops originally described as the ‘ferruginous clay of the upper stage of the Molasse formation’. They are overlain by light grey clays and underlain by marl [22]. All layers can be correlated to the Khersonian (i.e. Late Sarmatian s.l.) (9.9−7.6 Ma). The stratigraphy is based on the similarity of the layers with Mt Mithridat (see below) and needs modern revision. Direct information about dating is absent. We consider this locality as a type locality since Eichwald [22] mentioned that illustrated bones from his publication were found there.

*Other localities and ages.* All accessible or illustrated records positively identified by us as *Monachopsis pontica* were dated to the Khersonian or Maeotian (a more precise stratigraphic assignment is currently impossible) and are restricted to the Kerch Peninsula (eastern Crimea, Ukraine) and Küçükçekmece (Istanbul, Turkey) (figure 3). They include seven localities:

1. Khroni Cape (Crimea, Ukraine; also known as Khronya and referred to as the villages of Osoviny and Jurkine (or Yurkine); approximate geographical coordinates 45.439, 36.581). The locality is rich in *M. pontica* bones. The part of outcrops on the Khroni Cape bearing the specimens belong to the upper Khersonian (9.9−7.6 Ma) and lower Maeotian age [9]. The specimen TNU CH00-01 was found *ex situ* on a beach, whereas FFM 10246 was reportedly found *in situ* in the clay deposits.
2. Mt Mithridat, Kerch, Crimea, Ukraine (geographical coordinates 45.350, 36.469). Layers with seals have the same original description as layers of the Ak Burun [22]. They also overlay with grey clays which include *Chersonimactra caspia* and lay on marl (which also includes *Chersonimactra caspia*) [13]. So, all these layers can be correlated to the Khersonian (9.9−7.6 Ma).
3. Kamysh-Burun (Crimea, Ukraine; specifically quarry E, approximate geographical coordinates 45.283, 36.400). This is a Khersonian outcrop with *Chersonimactra caspia* remains, which are overlain by Cimmerian (Pliocene) deposits [12]. The context of the findings is unknown.
4. Töbeçik (Crimea, Ukraine; approximate geographical coordinates: site 7—45.153, 36.398; site 10—45.168, 36.333)—which includes two fossil localities with findings of *M. pontica*. Site 7 probably is the Khersonian [50], while site 10 correlates to the Bessarabian–Khersonian boundary or the lowest Khersonian. All the specimens were found *ex situ* on a beach.
5. Kyz-Aul (Qız-Aul) (Crimea, Ukraine; also known as the Providence quarry, the Belgian quarry or the Old French quarry, approximate geographical coordinates 45.083, 36.350). The section has the Khersonian deposits overlain by Cimmerian (Pliocene) layers containing iron ore [58]. The context of the findings is unknown.
6. Uzunlar (Crimea, Ukraine; approximate geographical coordinates 45.075, 36.107). Identical to the previous one, this section has a Khersonian age overlain by Cimmerian (Pliocene) layers containing iron ore [58]. The context of the findings is unknown.
7. Küçükçekmece (Istanbul, Turkey; approximate geographical coordinates 40.989324, 28.775374). According to Malik & Nafiz [24], seals were found in Khersonian layers that included *Chersonimactra caspia* and *Chersonimactra bulgarica.*

Most of these localities need modern dating.

### Emended diagnosis

*Monachopsis pontica* differs from all known phocine seals by having a thin posterior part of the nasal (as wide as 7.5% of the rostral width); an elongated maxillary part of the rostrum with a DNFO/RH ratio of about 195% (distance from nasal foramen to orbit/height of the rostrum at the rostral edge of the nasal; see figure 1); and a ventral edge of the zygomatic arch in anterior view situated higher than the alveolar plane (rather than a level with that plane). Also, it is distinct in a very long deltoid crest of the humerus reaching the coronoid fossa [11]. The long deltoid crest is shared only by several Monachinae seals such as *‘Virginiaphoca magurai’* (holotype humerus USNM 639750), *‘Auroraphoca atlantica’* (holotype humerus USNM 181419), and *Homiphoca*, and a phocine seal *Cryptophoca maeotica* (humerus NMNHU-P 64-530), from the Paratethys as well. *M. pontica* has a distally (rather than proximally) situated *pronator teres* process of the radius.

Also, *Monachopsis pontica* differs from all known phocine seals except *H. fasciata* in pm4 size, which is about equal to m1. Further, it differs from all known phocine seals except *H. fasciata* and *Pusa sibirica* by having a swollen palatal process of the maxilla [11]. It differs from all phocine seals except *H. fasciata*, *Pagophilus groenlandicus*, and *Erignathus barbatus* in a greater tubercle of the humerus higher than a head (see also [11]). Meanwhile, *M. pontica* shares the following synapomorphies with crown phocine seals, differing herewith from all the known Neogene phocines: strong inflation of a tympanic bulla; a lesser tubercle of the humerus higher than a head (see also [11]); a strongly twisted wing of the ilium (not shared by *Cystophora cristata* and *E. barbatus)*; and a strongly reduced intertrochanteric crest of the femur (not shared by *E. barbatus*).

New findings show that the PM4 and M1 are double-rooted, and the diastema between them is present (the observation differs from diagnoses made by [11]).

### Species diagnosis

The same as for genus.

## 3. Description

*Key points of the original description.* Eichwald [22] described the bones originating from the Khersonian layers of the Kerch Peninsula, that description focusing on general morphology. However, Eichwald [22] mentioned the long and narrow nasal bones (not illustrated) and the molar teeth with obtuse triangle crowns and thick (4.5 mm) and short (6.8 mm) roots.

*Body size.* Based on direct measurements of FFM 10246, the length of the body (from the snout to the end of the tail) is estimated as 75−80 cm. The estimation of body length is based on power regression [59]:


$$y=30.989x^{0.1365}-50.658,$$

where *y* is the length of the humerus (in cm) and *x* is the body length (in cm).

For the humerus of FFM 10246 (5.9 cm greatest length), an estimated body length is 81 cm and for the largest available bone NMNHU-P 64-248 OF 1008 (7.8 cm) the estimated body length is 104 cm (see electronic supplementary material, table S4).

*Skull.* The skull TNU CH00-01 includes the rostral part (premaxilla, maxilla and nasal), the infraorbital part (frontal, lacrimal and palatine), the anterior end of the jugal, and the roots of a few maxillary teeth. The anterior portion of the skull TNU CH00-01 (from the anterior tip of the rostrum to the braincase) is 9.7 cm in length. The rostrum is narrower and more elongated than in modern Phocidae. The lateral angle bordering the facial area of *M. pontica* (between the line of tooth row and nasal bone) is ~25°. The dorsal angle of the rostrum (between lateral lines of the maxilla from the dorsal view) is ~36° (electronic supplementary material, figure S2). In the lateral view, the border of the nasal cavity is concave. The skull has just barely seen inter-maxillary and inter-frontal sutures so it belongs to a cranially mature animal (figure 1; electronic supplementary material, figure S2) [60].

The *maxilla* of TNU CH00-01 has swelling on the lateral side. The infraorbital foramina are shifted posterolaterally, and the caudal edge of the foramen is visible in the dorsal view, its major axis is 5.5 mm and located horizontally, while the minor one is 3.7 mm. The infraorbital canal is positioned at the level of the M1. The palatine process forms are ventrally convex, expand posteriorly at the level of the first premolar and form a convex palate surface with a concavity near the midline. The palatine fissure is located at the level of the canine. The interincisive suture and palatal groove of the maxilla are strongly pronounced. The palatal groove is more pronounced near the anterior palatal foramen, which is located at the level of the M1. The lateral groove is shallow. The nasolabial fossa is narrow and deep and is situated between the levels of the PM4 and caudally to the M1 on the antorbital process of the maxilla.

The *nasal* bone is thinner than the minimum interorbital width and expands wider at its rostral third. Its posterior end is situated posterior to the infraorbital canal. The bone reaches the anterior quarter of the interorbital region (TNU CH00-01). The posterior end of the nasal bone is pointed.

*Frontal.* The interorbital area of the skull is narrow, its least width is 6.6 mm and located in the anterior part of the interorbital area. The supraorbital process of the frontal is not developed.

*Jugal.* The anterior end of the jugal is located at the level of the diastema between the M1 and PM4.

**Figure 5.**
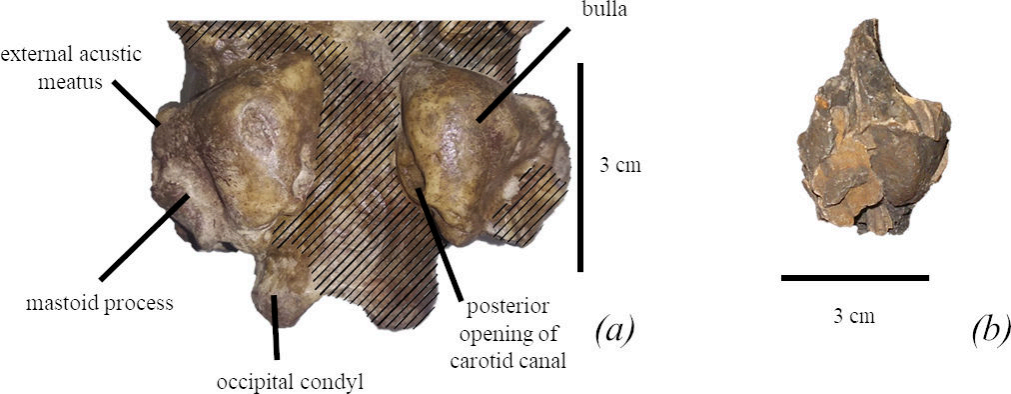
Bullae of *M. pontica.* (*a*) Basicrania of FFM 10246. Hatching—reconstruction. (*b*) Auditory bulla from MPGI 113 collection.

*Tympanic bulla.* The tympanic bulla of FFM 10246 has strong inflation, and the posterior opening of its carotid canal is weakly visible in the ventral view (figure 5). An external cochlear foramen is absent. Ventrally petrosal is not visible at the bulla–basioccipital junction.

*Mandible.* In the dorsal view, the mandible FFM 10246 is straight. In the lateral view, the body of the mandible gradually heightens from the anterior end. It reaches its maximum height (20 mm) at the level of the pm4, and posterior to it becomes slightly narrower. The chin prominence is developed. The second mental foramen is under the first root of the pm2 and the third mental foramen is under the pm3. The coronal process and other proximal parts of the bones were not preserved.

*Dentition. M. pontica* has three upper incisors. The number of the lower incisors is unknown. Each jaw has a canine, four premolars, and a single molar. Premolars oriented parallel to tooth rows. The maxilla (NMNHU-P 64-516) has a reduced diastema between premolars (one-third of PM root), while diastemas of TNU CH00-01 are better pronounced (two-thirds of PM root). The diastema between PM4 and M1 in both specimens is about twice as big as the diastema between PM3 and PM4.

**Figure 6.**
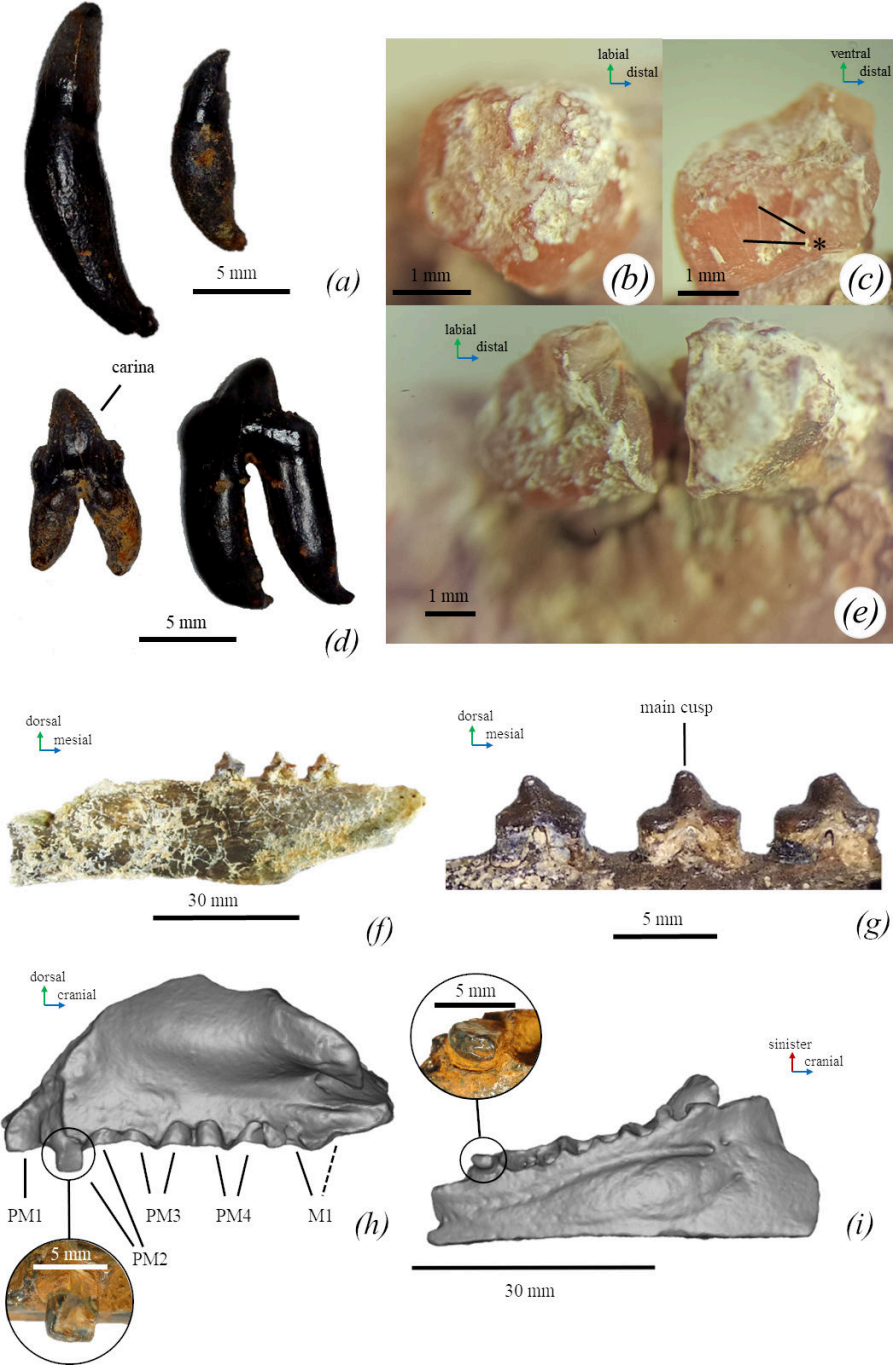
Dentition of *M. pontica*. (*a*) A canine and the third incisor TNU CH05, distal view. (*d*) Second and, probably, fourth premolars TNU CH05 of *M. pontica,* labial view. (*b,c,e*) Wearing of teeth of *M. pontica* (TNU CH00-01) (made by Svitozar Davydenko). (*c*) The enamel surface of the distal root of the PM3. (*d*) The enamel surface of the mesial root of the PM4, lingual view. (*e*) The enamel surface of the PM4. (*f*) The mandible FFM 10246, medial view. (*g*) Third and fourth premolars, and a lower molar (FFM 10246, medial view). (*h*) The maxilla of *M. pontica* NMNHU-P 64-516 with the zoomed root of the PM2** (lateral view). (*i*) The maxilla of *M. pontica* NMNHU-P 64-516 with the zoomed root of the PM2** (ventral view). *Damage is *post-mortem*. **Photo made by Dmitry Ivanoff.

*Teeth.* Based on the available maxilla of *Monachopsis pontica* (CH00-01), the alveoli of the third incisors are present and are 1.3 times larger than the alveoli of the second one. The incisor alveoli are transversely compressed. The canine alveolus diameter is twice as large as the alveoli of the third incisor. The first maxillary premolar is single-rooted and slightly smaller than PM2. The second to fourth premolars and the molar are double-rooted. The fourth premolar and molar have similar sizes. The M1 and PM4 are also similar in alveoli size (figure 6).

According to Koretsky [56], the PM4 and M1 of NMNHU-P 64-516 are single-rooted. However, reviewing that material, we found that the remaining tooth root belongs to PM2 rather than to PM1, and the PM4 is double-rooted. While the alveolar process of the M1 is partially broken on the area of the probable position of the second root, it is unclear if the M1 is single-rooted or double-rooted (figure 6). Therefore, both NMNHU-P 64-516 and CH00-01 maxillae directly show that *M. pontica* had double-rooted PM4.

The tooth crowns of the mandibular premolars and the mandibular molar of the specimen FFM 10246 have a cone-shaped main cusp. The main cusp is located laterally on the teeth crown and is more than twice as small in length as the tooth crown (see electronic supplementary material, table S6) and lies on the flat plateau. The lingual cingulum is absent and the enamel is rather smooth (figure 5). The isolated premolars of TNU CH05 have a similar shape. The second premolar of TNU CH05 has carinae on both mesial and cranial edges.

*Tooth wear.* Some skull fragments of *M. pontica* possess teeth worn to roots. TNU CH00-01 has posterior roots with the worn surface of the left PM2 and PM3, both roots of the left PM4, and the anterior root of the right PM4. All the worn surfaces are on the same horizontal level, which suggests that they were worn during the lifetime of the animal but were not damaged in such a way during fossilization. However, the tooth enamel shows some damage (cracks and small round chops on the surface of the enamel) that has been caused, probably, due to taphonomic processes. Because of these damages, it is difficult to state if any in-life scratch marks are present (figure 6). The only remaining tooth root of PM2 of NMNHU-P 64-516 also has a flat surface (figure 6).

*Vertebrae* (FFM 10246; figure 2).

*Cervical.* The transverse foramen of the atlas is visible in the posterior view, and the direction of the transverse process in the lateral view is oblique. The axis has a well-developed neural process, which becomes higher in a caudal direction. The spinous and transverse processes of other cervical vertebrae are small.

*Thoracic.* The spinous processes of anterior thoracic vertebrae are long (up to 1.8 times longer than the width of the centrum). The transverse processes are laterally directed. Posterior thoracic vertebrae have low spinous processes (about 60% of the width of the centrum). Their transverse processes are directed caudally.

*Lumbar.* The spinous process of the lumbar vertebrae is low (not higher than the height of the centrum).

*Sacrum.* Both sacra TNU CH00-02 and FFM 10246 have similar shapes to the sacrum of modern phocines and consist of four fused vertebrae. The sacrum TNU CH00-02 is almost complete except for the broken medial crest. The spinous processes of CH00-02 are completely fused, its wings are slightly directed in cranial direction (110° to the line of the body) (lectrinic supplementary material, figure S3), the lateral crest is about the same width at the widest point of every vertebra (first: 22.9 mm, second: 23.9 mm, third: 22.8 mm and fourth: broken), whereas in FFM 10246 the fusion is strong but not complete. The wings are slightly cranially directed (100° to the longitudinal axis of the body). Its lateral crest is much wider at the level of the second sacral vertebra than it is at the level of the first and third ones (first: 26.8 mm, second: 31.79 mm, third: 25.6 mm and fourth: 19.6 mm). Both specimens have sacral wings as wide as ~110% of the first sacral centrum width.

**Figure 7.**
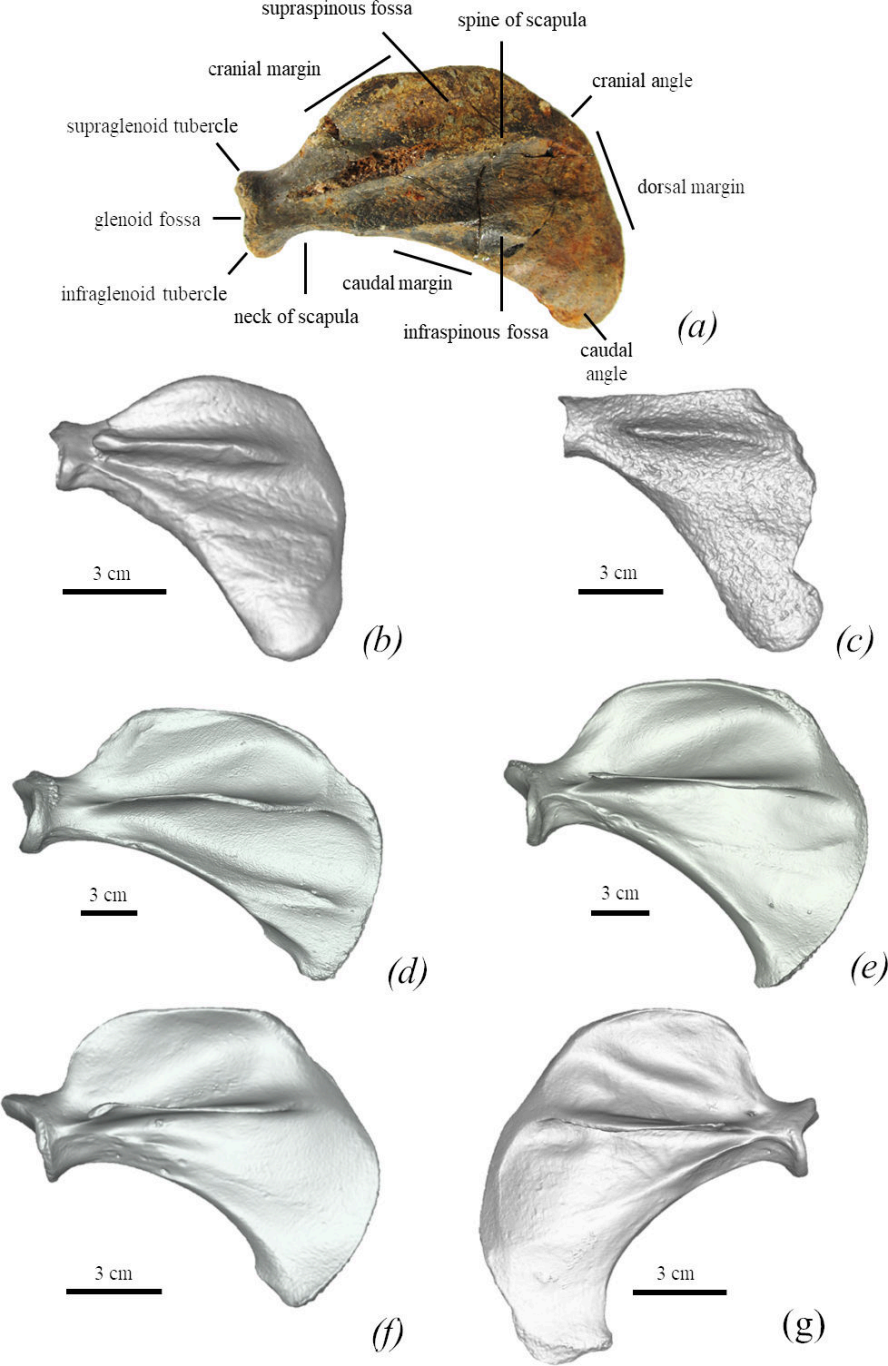
Comparison between seal scapulae of different species. All figured in lateral view. (*a*) *M. pontica* TNU CH05. (*b*) *M. pontica* FFM 10246. (*c*) *Pachyphoca* sp. NMNHU-P 64-477. (*d*) *E. barbatus* NWM 1950. (*e*) *Ha. grypus* NWM 31589. (*f*) *Ph. vitulina* NWM 1462. (*g*) *Pu. caspica* NWM 66298.

*Scapula.* The neck of the scapula (TNU CH05) is approximately 90% of the width of the articulated surface. The coastal surface of the scapula is almost a semicircle but has an elongated dorsal margin, which has a rounded edge. The cranial margin gradually expands and is twice as high as the supraglenoid tubercle if the measurement is based on the spine of the scapula. The cranial margin smoothly transits into the dorsal margin. The caudal margin is smooth and ventrally concave. The scapular spine is weakly developed. The infraspinous fossa is twice as large as the supraspinous fossa. The infraspinous fossa is elongated caudally. The length of the scapula is nearly the same as the width of the scapular blade. The lateral ridges are not joined near the glenoid part (figure 7).

**Figure 8.**
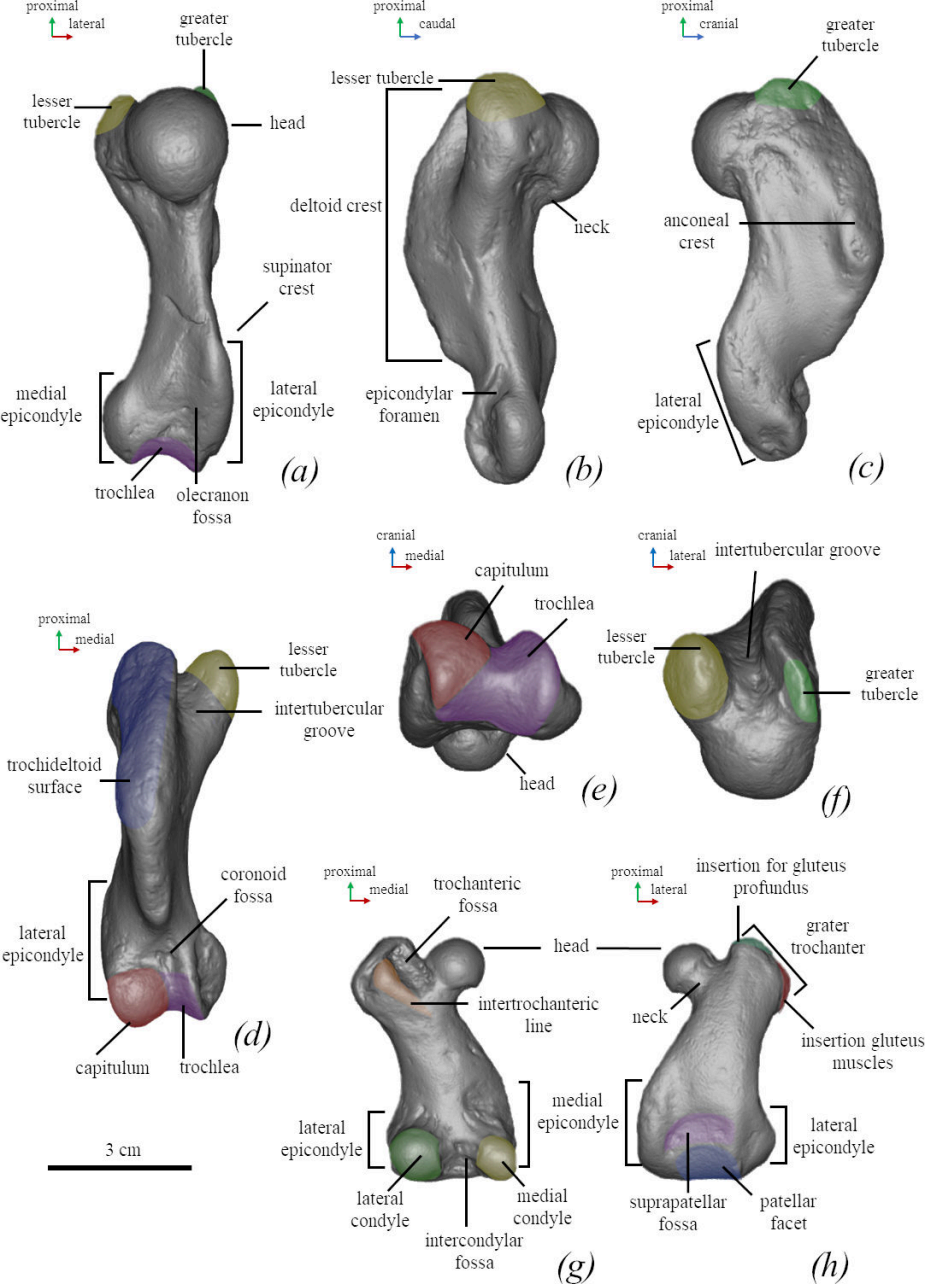
Anatomical nomenclature of the humerus (NMNH-P 64-257) (*a–f*) and femur (*g,h*) of *M. pontica* (NMNH-P 64-256). Digital models of the bones: (*a*) posterior, (*b*) medial, (*c*) lateral, (*d*) anterior, (*e*) inferior, (*f*) superior, (*g*) posterior and (*h*) anterior views.

*Humerus* (FFM 10246). The humerus is robust (the smallest width of the diaphysis is 15% of the bone length) with an S-shaped outline from the lateral view. The deltoid crest is well developed and its length corresponds to the three-fourths of the bone. The humeral head is spherical and robust, whereas the neck is short. The transition between the humeral head and the shaft is ventrally concave and dorsally smooth. The lesser tubercle is robust, its proximal end is below the proximal end of the greater tubercle and slightly higher than the head. The proximal bifurcation of the deltoid crest is not developed. The intertubercular groove is narrow and deep. The lateral epicondyle is massive and measures around one-third of the bone length. The medial epicondyle is slightly medially developed. It is twice as short as the lateral one. The entepicondylar foramen is present and fully penetrates the medial epicondyle. The supinator crest projects slightly dorso-caudally. The width of the caudal surface of the trochlea is similar to the capitulum (figures 4 and 8). As shown by Dewaele *et al*. [61], the humerus is highly compact and is pachyosteosclerotic in anatomy.

**Figure 9.**
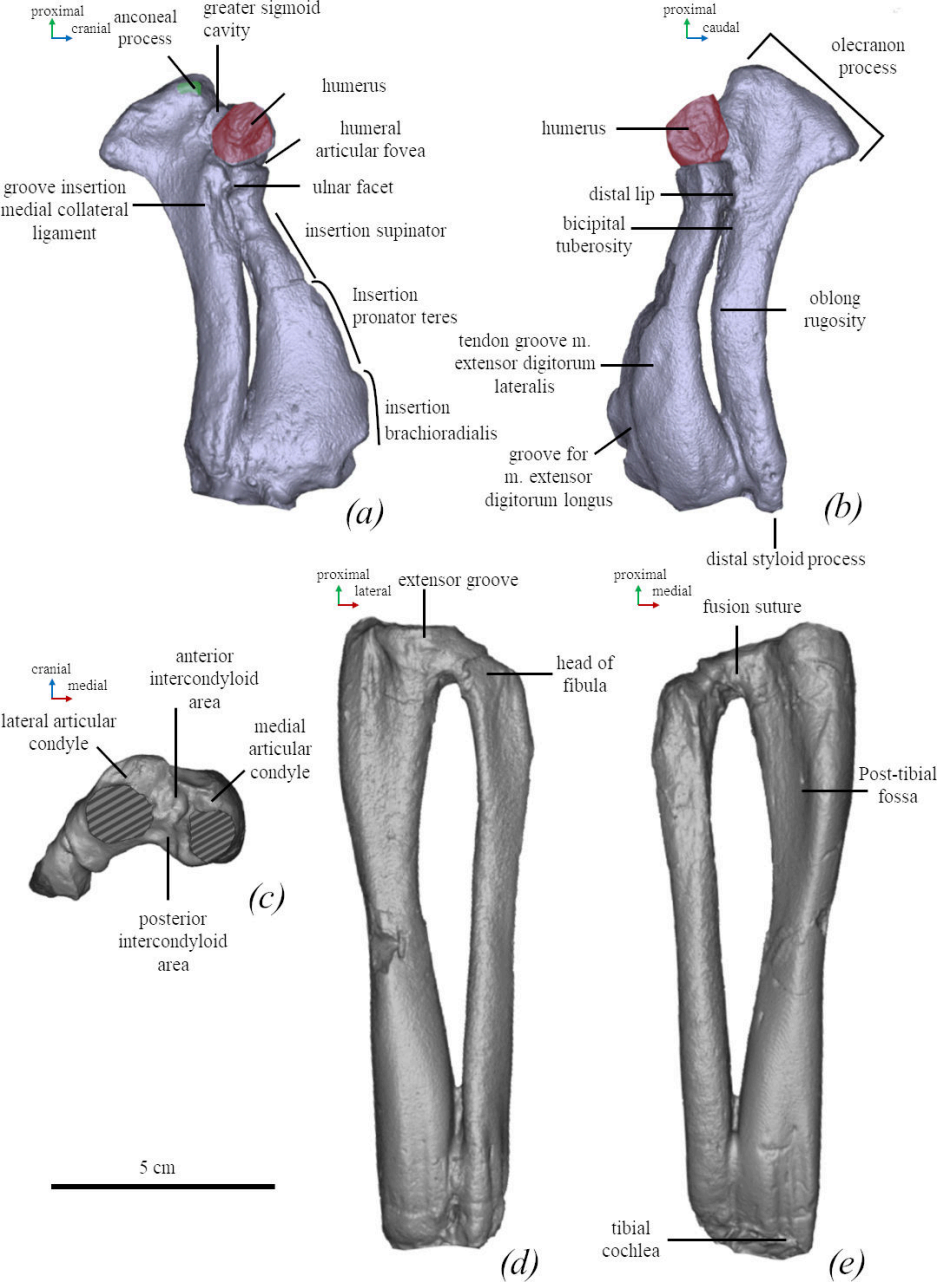
Anatomical nomenclature of the left ulna and radius (*a,b*) and tibia and fibula (*c–e*) of *M. pontica* (FFM 10246). Digital models of the bones: (*a*) medial, (*b*) lateral, (*c*) superior, (*d*) anterior and (*e*) posterior views.

*Radius* (FFM 10246). The cranial edge of the radius is rounded. The neck of the bone is curved. The radial tuberosity is placed on the posteromedial side. The *pronator teres* process is located proximally. The proximal edge of the insertion *brachioradialis* starts at the level of 35% of the bone length (from the bone’s distal edge). The distal part is wide (2.5 times wider than the radial neck) (figure 9).

*Ulna* (FFM 10246). The olecranon has a straight proximal edge and is placed at a 60° angle to the diaphysis. The olecranon is 2.4 times wider than the diaphysis. The width of the diaphysis is almost equal throughout the entire length of the bone. The trochlear notch covers around 15% of the whole bone length. The styloid process is pointed (figure 9).

*Metacarpal I* (FFM 10246). The bone is bent in a cranial direction and flattened in latero-medial direction. The bone is slightly longer than metacarpal II (metacarpal I is 115% of metacarpal length).

*Pelvis* (FFM 10246). The ilium is a short bone and is only 1.5 times longer than the acetabulum. Its cranial edge is round, and its dorsal portion is slightly smaller than the ventral one. The gluteal fossa is shallow. The wing of the ilium is moderately bent laterally from the main axis of a coxae. The iliopectineal eminence is moderately developed (electronic supplementary material, figure S3).

**Figure 10.**
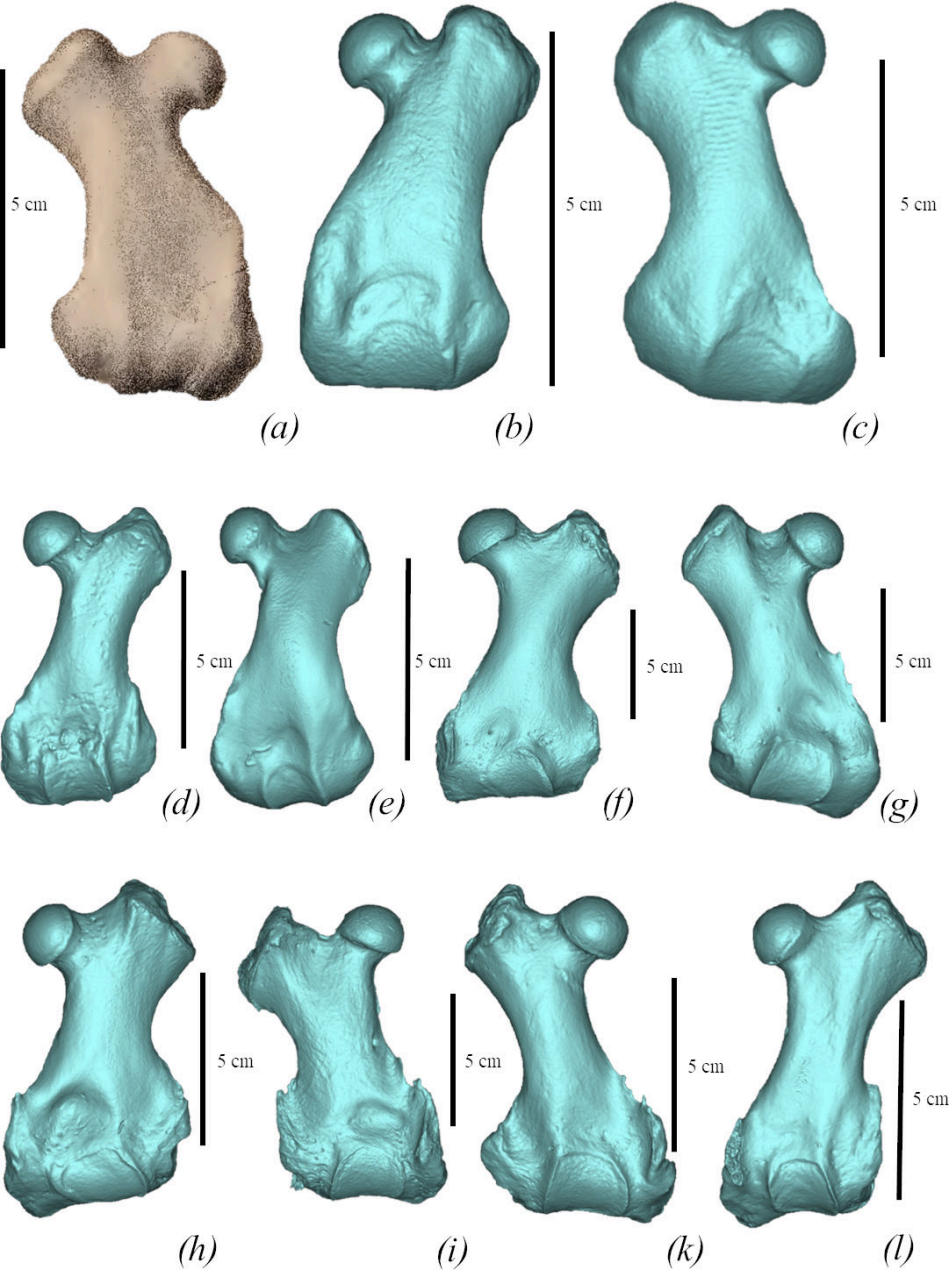
Comparison of femora of true seals (Phocidae) in cranial view. (*a*) *M. pontica* MPGl 25-113. (*b*) *M. pontica* NMNHU-P 64-256. (*c*) *M. pontica* NMNHU-P 64-314. (*d*) *Cr. maeotica* Nordman (no. 1). (*e*) *Cr. maeotica* NMNHU-P 64-455. (*f*) *E. barbatus* CN 958 (NHMD). (*g*) *C. cristata* 1134 (NHMD). (*h*) *Pa. groenlandicus* 154 (NHMD). (*i*) *Ha. grypus* 1485 (NHMD). (*k*) *Ph. vitulina* NMBE 301 91. (*l*) *Pu. caspica* NMW 66299.

*Femur* (FFM 10246). The femur is short and wide (56.8 mm in length, the smallest width in the anterior view 18.2 mm and the largest 34.6 mm). It is twice shorter than the tibia. The neck of the femur is short, narrow and well separated from the head. The neck and head width ratio equals 0.6. The greater trochanter is short and robust. The head is about on the same level as the greater trochanter. The head is orientated medio-distally. The trochanteric fossa takes about half of the greater trochanter. The intertrochanteric crest is low. The medial margin of the greater trochanter and the diaphyseal medial margin are in the same plane. The medial edge of the diaphysis is almost straight and smoothly transits into the medial epicondyle. The medial epicondyle is about 40% of the bone length and the lateral one is about 30%. The lateral edge of the diaphysis is moderately concave. The smallest width of the femoral diaphysis measures at least half of the proximal or distal epiphyseal width. The proximal and distal epiphyses have almost equal width. The width of the medial condyle measures three-fourths of the width of the lateral condyle. While the lateral epicondyle projects in the lateral direction. The suprapatellar fossa is present and shallow (figures 8 and 10 and electronic supplementary material, figure S4).

**Figure 11.**
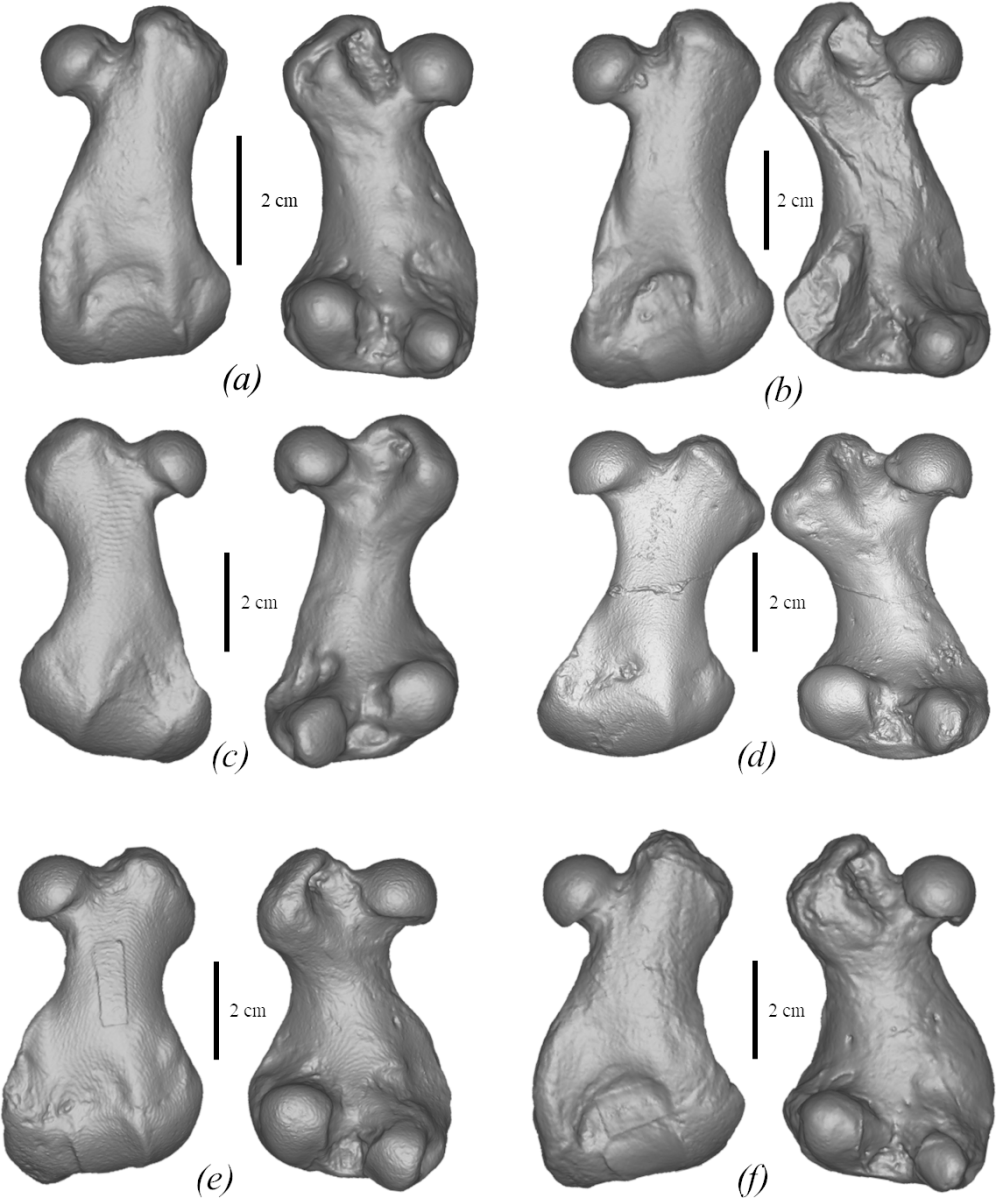
Variability of the femur of *M. pontica*. (*a*) NMNHU-P 64-256, (*b*) NMNHU-P 64-254, (*c*) NMNHU-P 64-314, (*d*) NMNHU-P 64-457, (*e*) NMNHU-P 64-361, (*f*) TNU CH4a-029.

Two femoral morphotypes are present at all sites where *M. pontica* femora were found in large numbers, such as Kamysh-Burun and the Khroni Cape. No other seal taxa have been recorded from those localities. Therefore, we propose they both belong to *M. pontica*. Two morphotypes can be distinguished by the medial edge of the diaphysis and the medial epicondyle. The morphotype 1 is characterized by a mostly straight medial line (NMNHU 64-314, 64-457, 64-254; figure 11*a−d*). In the morphotype 2, the medial line is convex in the proximal–medial direction (64-256, 64-631, FFM 10246, CH4a-029) and the medial epicondyle is more massive than the morphotype 1 (figure 11*e,f*).

*The tibia and fibula* FFM 10246 are fused in the proximal part (distally the bones are not fused). Both bones are elongated. The tibia is slightly longer (108%) than the fibula. A post-tibial fossa is well developed. The tibia is slightly curved in the proximal half, and almost straight distally. The fibula is flattened in the cranial–caudal direction (figure 10).

**Figure 12.**
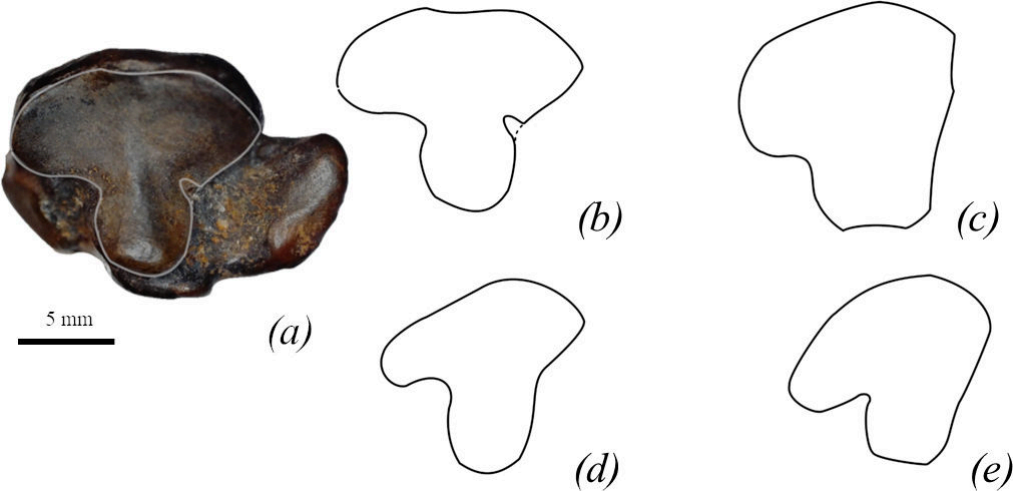
Comparison of cuboid bones of true seals (Phocidae). (*a*) CH05 *M. pontica* cuboid in distal view with the highlighted edge of the facet for the fourth and fifth metatarsals. (*b–e*) Outlines of facets for the fourth and fifth metatarsal. (*a*,*b*) *M. pontica* CH05*,* (*c*) *C. cristata* USNM-550317, (*d*) *E. barbatus* UWBM-34220, (*e*) *Ha. grypus* USNM-504481. (*c*–*e*) Redrawn from virtual.imnh.iri.isu.edu.

*Cuboid.* The facet for the fourth and fifth metatarsal is T-shaped (figure 12).

## 4. Comparison

*M. pontica* belongs to Phocidae as supported by the reduced premaxilla-nasal suture (character no. 1 in this study; see electronic supplementary material), posterior location of the anterior opening of the infraorbital foramen to M1 (no. 10), mortised squamosal–jugal articulation (no. 13), low interorbital least width (among the narrowest in Phocidae, no. 18), a paroccipital process well separated from the mastoid (no. 27), no lingual cingulum of postcanine teeth (no. 38), absent lesser trochanter of the femur (no. 68) and large differences between the size of distal condyles of the femur (no. 71).

*M. pontica* belongs to Phocinae by the presence of maxilla swelling (no. 7), the anterior position of interorbital least width on the interorbital septum (no. 17), strong development of humeral supinator (no. 47), present entepicondylar foramen of the humerus (no. 53), metacarpal I and II are about the same in size (no. 58), and four fused vertebrae in the sacrum (no. 60) [62,63].

**Figure 13.**
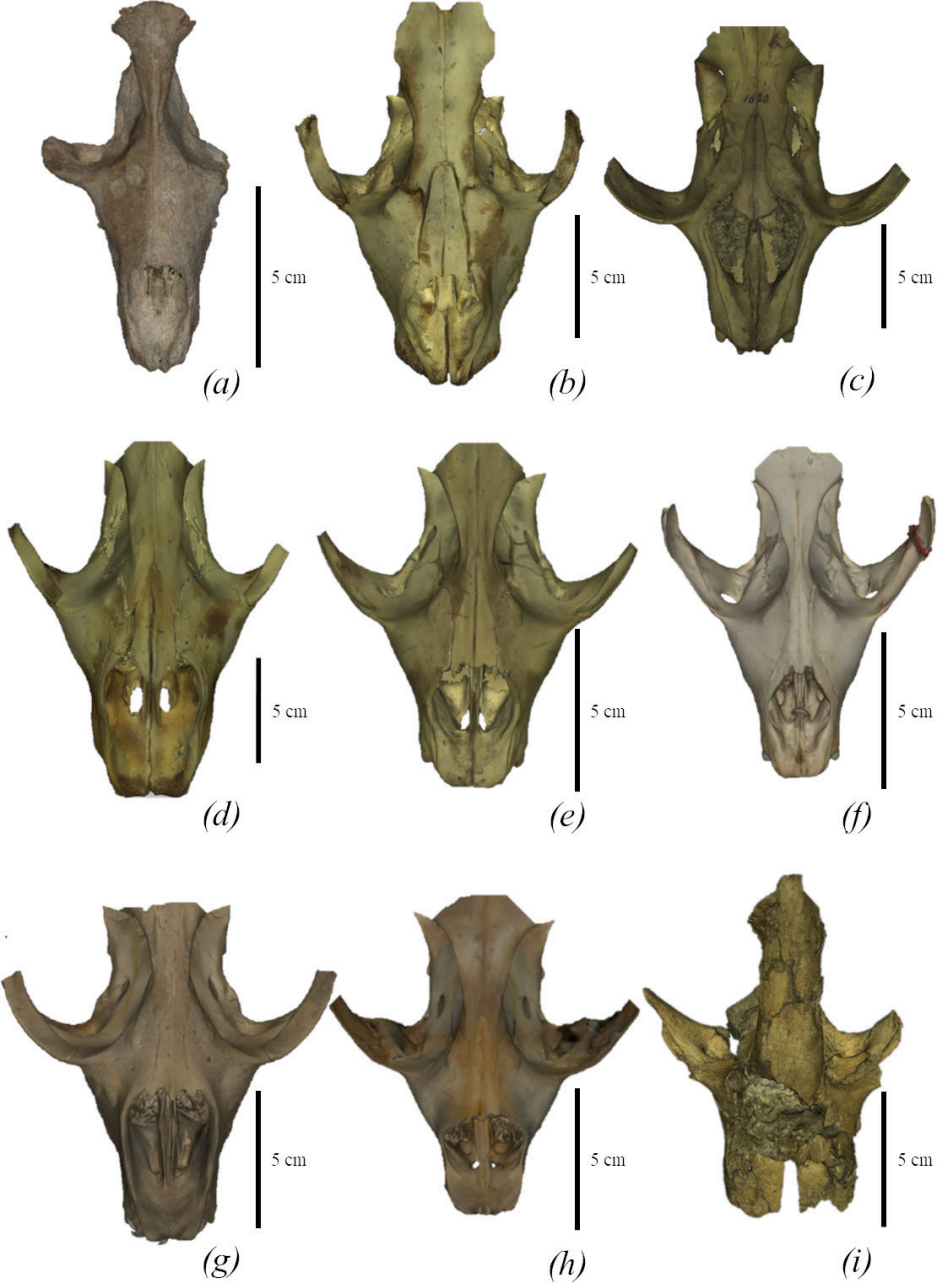
Comparison of seal skulls in dorsal view. (*a*) *M. pontica* TNU CH00-01. (*b*) *E. barbatus* NWM 4026. (*c*) *C. cristata* NWM 1620. (*d*) *Ha. grypus* NWM 31588. (*e*) *Ph. vitulina* NWM 1462. (*f*) *Pu. caspica* GNM 2-2013/98. (*g*) *Pa. groenlandicus* NMBE 638. (*h*) *H. fasciata* ZMUC CS-303-67. (*i*) *D. emryi* SNM Z27870.

*Monachopsis pontica* can be distinguished from all other Phocinae by the following unique traits: a thin posterior part of the nasal (7.5% of the nasal length while the nasal of other Phocinae is wider than 14%) (figure 13 and electronic supplementary material, table S7); *M. pontica* has a very long deltoid crest (80% of the humerus length) of the humerus, which reaches the coronoid fossa (electronic supplementary material, table S2). This feature is rare among true seals and is only seen in a few Monachinae (e.g. *‘Virginiaphoca’*, *‘Auroraphoca’* and *Homiphoca*) and such Paratethyan seals as *Cryptophoca*.

Further, *M. pontica* differs from most Phocidae, except *Pu. sibirica*, *H. fasciata and Pa. groenlandicus*, by having a convex palatal process of the maxilla. While in other Phocidae it is concave (character no. 90). *M. pontica* has a suprapatellar fossa of the femur. While other Phocidae usually do not have it (except *Pa. groenlandicus*, *Ha. grypus*, and *Pu. caspica, ‘Praepusa vindobonensis’, Nanophoca vitulinoides, Pliophoca etrusca*) (character no. 77) (see Diagnosis).

Also, among Phocinae (except *H. fasciata*) *M. pontica* can be distinguished by the same size of the pm4 and m1, while most Phocinae species have the pm4 larger than m1 (character no. 39).

### 4.1. Comparison with fossil taxa

For comparison with fossil species, we focused on Paratethyan true seals. Most Paratethyan seals were described based on isolated postcranial materials and their species referrals were based on ecomorphotype hypotheses [11], common locality and age. However, some recent studies [19,21] criticized this approach for the lack of significant statistical proof stating that correlation in the morphology of femora and humeri is not enough to suggest combining them in one species. Thus, following these studies, the species/genera with the type material that includes only isolated postcranial bones, as is the case in most Paratethyan species, were considered *nomina dubia.* However, there are at least six morphotypes of humeri and six morphotypes of femora in the Central and Eastern Paratethys records that display morphologically distinct autapomorphic characters. Those forms have been assigned to the genera *Monachopsis*, *Praepusa*, *Cryptophoca*, *Pontophoca*, *Devinophoca* and *Pachyphoca* [11,18,22,64–68]. The lack of cranial and/or associated materials for some of these groups makes a revision of these taxa challenging. However, in their revisions, their diagnostic postcranial elements should be considered. So, for this study, we used previously erected names and features of postcranial bones to highlight and distinguish various types of femoral and humeral morphological sets from each other and, more importantly, from *M. pontica*. We emphasize that each of the Paratethyan seal taxa requires careful case-by-case taxonomic revisions based on the diagnostic characteristics and ‘context’ of the type materials (whether the types are postcranial or cranial elements). For comparison, in this study, we selected specimens with the most complete fossil material if it was possible.

#### *Devinophoca* spp.

*D. emryi* (SNM Z27870–Z27873), *D. claytoni* (Z14523), and the postcranial material found in the same locality (SNM Z14543, Z14544, Z25507, Z27874, SNM PP1502, PP1503). These seals are known from the Middle Miocene of the Central Paratethys (Bonanza Hill, Slovakia) [67,69,70]. Both species (*D. emryi* and *D. claytoni*) were found and described from the same location (Bonanza Hill), where no other seals have been recorded from. Both *D. claytoni* and *D. emryi* were described based on the skulls. All the described postcranial bones have been found in the same excavation as the skull of *D. emryi* in 1997−1998 and later were assigned to *D. emryi* [67]. Therefore, we compared these materials as pooled from two nominal species.

*M. pontica* has a thinner and longer rostrum (137% of the interorbital septum length), and the dorsal side of the rostrum is straight from the lateral view. *M. pontica* has a deep nasolabial fossa. The tooth row is straight; *M. pontica* has two roots of M1 and developed the main cusp of pm3. Whereas *Devinophoca* has a wider and shorter rostrum (107% of the interorbital septum length), and a slight hump in the infraorbital region of the rostrum (figure 13; electronic supplementary material, figure S2), *Devinophoca* has a shallow nasolabial fossa. The tooth row is curved (electronic supplementary material, figure S5); M1 of *Devinophoca* has three roots, and pm3 has moderately developed mesial and distal accessory cusps.

Further, *M. pontica*: has a short humeral diaphysis (diaphysis/head ratio is 2.35) (figure 3; electronic supplementary material, figure S6); the femoral head-to-greater trochanter distance is smaller than the medial-to-lateral epicondyle distance. The distal epiphysis is larger than the proximal one (its width is 93% of the distal one). *M. pontica* has a shorter and lower olecranon of the ulna (30% of the bone length). Humeri SNM PP 1502 and Z25507 have a long humeral diaphysis (diaphysis/head ratio is 3.4), the femur SNM Z14544 has a head-to-greater trochanter distance larger than the medial-to-lateral epicondyle distance, the proximal epiphysis is larger than the distal one (the width of the proximal epiphysis is 110% of the distal one width). The ulna SNM PP1503 has longer and higher olecranon (36% of the bone length).

In the lateral view, the ilium of *M. pontica* is 1.5 times larger than the diameter of the acetabulum. The coxae SNM Z14543 has a longer ilium, which is 2.4 times larger than the acetabulum.

#### *Praepusa* spp.

This taxon includes 6 nominal species, most of which were suggested as *nomina dubia* [21]. Therefore, here we operated only with the most complete available specimens of *Praepusa* NMNHU-P 64-468, 64-469 containing skull material. Specimens were first described by Antoniuk and Koretsky (1984) as *Praepusa tarchankutica* and later assigned to *Praepusa vindobonensis* by Koretsky [11]. Here we used their original name to discuss their comparison with *M. pontica*, whereas their full revision goes beyond the scope of this paper.

#### *Praepusa tarchankutica* (NMNHU-P 64-468, 64-469)

A Paratethyan seal known from the Bessarabian (latest Middle and early Late Miocene) of the Crimean peninsula, Ukraine [11,71].

*M. pontica* has a longer rostrum (137% of the interorbital septum length) than *Pr. tarchankutica* (130% of the interorbital septum length). However, both species have a deep nasolabial fossa and a deep depression under the infraorbital foramen. Other skull characters are impossible to compare due to the deformation of the *Pr. tarchankutica* skull NMNHU-P 64-468. *M. pontica* has only the main cusp of the pm2-m1, the mandible body of *M. pontica* reaches a maximal height at the level of pm4, while mesial and distal accessory cusps of *Pr. tarchankutica* are well developed and the maximal height of the mandible body is at the level of a diastema between pm3 and pm4. Greater and lesser humeral tubercles of *M. pontica* are higher than the head while tubercles of *Pr. tarchankutica* are lower than the head.

#### *Cryptophoca maeotica* (NMNHU-P 64-455, 64-530, NMNHU-P Nordmann unnumbered humerus, Nordmann (1) femur)

A Paratethyan seal known from the Bessarabian (latest Middle and early Late Miocene) of the Eastern Paratethys (Chișinău, Moldova; Kamysh-Burun, Ukraine; Töbeçik, Ukraine) [11,72].

*M. pontica* has a short humerus (medial length: 49−67.5 mm; for more details, see [11]), the distal end of the humeral deltoid crest has a sharp angulated edge while *Cr. maeotica* has a long humerus (100.8 mm for NMNHU-P 64-530 and 95.5 for NMNHU-P Nordmann unnumbered), and the distal end of the humeral deltoid crest has a smooth transition to the body (figure 4; electronic supplementary material, figure S6). *M. pontica* has a wide femoral diaphysis: the smallest width of the femur is larger than or equal to half of the width of the distal epiphysis, whereas the same width in *Cr. maeotica* measures less than half (NMNHU-P 64-455, Nordmann (1)). The femur morphotype 1 of *M. pontica* differs from *Cr. maeotica* in a more laterally situated distal part of the medial epicondyle (figure 10; electronic supplementary material, figure S4). The trochanteric fossa of *M. pontica* is shallow and not wider than half of the greater tubercle width. Whereas the trochanteric fossa of *Cr. maeotica* is deeper and wider than half of the greater tubercle width.

#### *Pachyphoca* spp. (NMNHU-P 64-166, 64-354, 64-481, 64-701, 64-710)

The genus includes two nominal species *Pachyphoca chapskii* and *Pachyphoca ukrainica* selected here for comparison. A Paratethyan seal known from the Bessarabian (latest Middle and early Late Miocene) of the Eastern Paratethys (Khomutove, Ukraine; Zhovtokamianka (Zheltokamenka), Ukraine; Zolota Balka, Ukraine) [18]

The humerus of *M. pontica* has a sharp angulated edge of the distal part of the deltoid crest, the insertion *brachioradialis* of the radius is shorter than the distal part of the bone, and an ulna has a well-developed olecranon, while the deltoid crest of *Pachyphoca* sp. (NMNHU-P 64-701) smoothly transits to diaphysis in the distal part, the insertion *brachioradialis* of the radius (NMNHU-P 64-481) is longer than the distal part, and the ulna (NMNHU-P 64-710) has slightly developed olecranon. The femoral medial epicondyle of *M. pontica* is shorter (about 40% of the bone length), and its femur has slightly developed trochanteric lines, while *Pachyphoca* sp. (NMNHU-P 64-354) has a long medial epicondyle (about half the length) and the trochanteric line of the femur is well developed.

#### *Histriophoca alekseevi* (NMNHU-P 40-121)

A Paratethyan seal known from the early Late Miocene of the Eastern Paratethys (Chișinău, Moldova) [11]. It was originally described by one rostral part of the skull and was suggested as *Phoca pontica* [23]. Currently, the material is considered lost or temporarily unavailable and cannot be directly revised but its illustrations can be discussed.

*M. pontica* has a short diastema between the canine and PM1 of the maxilla. Whereas, *H. alekseevi* has as wide a diastema as PM1. Also, the original description of this skull [23] includes nasal bones, which were lost before the redescription by Koretsky [11]. Alekseev [23] mentioned a narrow nasal, which became wider in the rostral part. This is similar to *M. pontica*. However, in Alekseev [23], an illustration of the skull in dorsal view is absent making it impossible to compare the shape of the nasal for this study.

#### *Planopusa semenovi* (NMNHU-P 64-709)

A Paratethyan seal is known as a rostral portion of the skull and a few teeth from Bessarabian deposits of Eastern Paratethys (Hrytsiv, Ukraine).

The ratio of the smallest width of the rostrum (at the level of PM 1) to the largest rostral width at the level of canine is larger in *M. pontica* than it is in *Pl. semenovi* (93% versus 86%). Usually, the difference in morphology between maxillary and mandibular postcanine teeth is low in Phocidae [73], so we suggest (with some caution, as some seals may have a lingual shelf only on the upper or lower teeth) that we may compare the mandibular teeth of *M. pontica* and the maxillary teeth of *Pl. semenovi. M. pontica* has only the main cusp which is located at the distance of mesial and distal ends of the tooth between tooth roots, its length is about 43–49% (for pm2-pm4) of the crown length, while in *Pl. semenovi* the distal accessory cusp is present in addition to the main one (moreover, in M1 mesial cusp is also present). The main cusp anteriorly reaches the mesial end of the tooth and is located mostly over the mesial root, its length is 59% of the crown length in PM4 and 50% in M1 [68].

### 4.2. Comparison with modern taxa

For comparison with modern taxa, at least one species per each Phocinae genus was available and/or included in this study.

#### *Erignathus barbatus* (NMW CN958, CN958, 7556)

*M. pontica* has a more elongated rostrum: DNFO/RH ratio of *M. pontica* is about twice as large as in *E. barbatus* (electronic supplementary material, table S7). *M. pontica* has a lateral angle bordering the facial area at 5° smaller and a dorsal angle of the rostrum at 9° smaller (figures 1 and 13) than these angles in the skull of *E. barbatus* (electronic supplementary material, table S7)*. M. pontica* has a straight nasal in the lateral view and the palate is convex, whereas *E. barbatus* has a convex nasal in the lateral view and the palate is concave.

*M. pontica* has a deep and narrow nasolabial fossa and does not have diastema between C, PM1 and PM2. In contrast, *E. barbatus* has a shallow and wide nasolabial fossa, and a diastema between C, PM1 and PM2 (electronic supplementary material, figure S5). Only the main cusp of pm2-m1 of *M. pontica* is developed whereas *E. barbatus* also has mesial and distal accessory cusps. The upper incisor alveoli of the teeth of *M. pontica* are strongly transversely compressed; they have an ellipse shape. The semi-major axis of I1 and I2 is 1.5 times larger than the semi-minor one. Contrary, *E. barbatus* has slightly transversely compressed alveoli of the teeth. The mandible body of *M. pontica* reaches the maximal height at a level of pm4 and the chin prominence is low, whereas *E. barbatus* mandible body reaches maximal height at a level of m1 and chin prominence is high.

In *M. pontica*, the highest point of the cranial margin is located about at the middle of the margin. The height of the dorsal margin is 60% of the bone length (at the level of the scapular spine), while in *E. barbatus* the highest point is located distally, and the height of the dorsal margin is 47%. The deltoid crest of *E. barbatus* has a huge trochideltoid surface. The medial epicondyle of the femur of *M. pontica* is 130% of the length of the lateral one, whereas the medial epicondyle of the femur of *E. barbatus* is slightly larger than the lateral (medial is 115% of the lateral one) (figure 10; electronic supplementary material, figure S4).

#### *Cystophora cristata* (ZMUC 1134, 1265)

The DNFO/RH ratio of *M. pontica* is about ten times larger than this ratio in *C. cristata*. The lateral angle of the face of *M. pontica* (figure 1) is 5° larger than that of *C. cristata,* while the dorsal angle of the rostrum is 21° less (figure 13; electronic supplementary material, table S7). *M. pontica* has the proximal edge of the nasal cavity on the level of PM2, the nasal is long and narrow (figure 13). The zygomatic process of the frontal is absent. *C. cristata* has the proximal edge of the nasal cavity on the level of the zygomatic arch. Its nasal is short and wide. The zygomatic process of the frontal is present. An external cochlear foramen is absent in *M. pontica* and present in *C. cristata.*

The tooth row of *M. pontica* has a rostrally directed angle. Its nasolabial fossa is deep and narrow. In contrast, the tooth rows of *C. cristata* are more or less parallel and the nasolabial fossa is shallow and wide. The cranial edge of the palate of *M. pontica* ends near the centre of the interorbital area. While in *C. cristata* it goes posteriorly to the centre of the infraorbital area. *M. pontica* has three incisors of the maxilla, and the I1 and I2 alveoli are strongly transversely compressed. The semi-major axis of ellipse-shaped alveoli is 1.5 times larger than the semi-minor one. In contrast, *C. cristata* has two maxillary incisors and its incisor alveoli have a rounded shape. *M. pontica* has developed a main cusp of pm3-m1, while *C. cristata* has a weakly developed main cusp. The roots of teeth are fused. The height of the *M. pontica* mandible slightly increases so it reaches a maximal height at the level of pm4 with a pronounced chin prominence. On the contrary, the mandible body of *C. cristata* is equally high and has a slightly outlined chin prominence.

The supraspinous fossa of the scapula of *M. pontica* is 27% of the bone length at the level of the scapular spine, while in *C. cristata,* it varies from 32% in male USNM-550317 to 39% in female USNM-188962.

*M. pontica* has a slightly developed supinator crest of the humerus in a posterior direction. The humeral lesser and greater tubercles are on the same level (figure 12; electronic supplementary material, figure S6). In contrast, *C. cristata* has a strongly developed supinator crest of the humerus in a posterior direction and the humeral lesser tubercle is higher than the greater one. The intertubercular groove of *M. pontica* is thinner than that of *C. cristata* (13% of the length of the humerus in *M. pontica* and 17% in *C. cristata*).

*M. pontica* has a much longer medial epicondyle of the femur than the lateral (the medial is 130% of the lateral one). In contrast, the medial epicondyle of the male *C. cristata* is slightly longer than the lateral one (the medial is 110% of the lateral one) and the female has medial epicondyle that is 150% of the lateral one.

#### Histriophocini

*M. pontica* does not have an external cochlear foramen, while all Histriophocini have it.

#### *Pagophilus groenlandicus* (skull NMBE 638, ZMUC 154, ZMUC CN 961)

The DNFO/RH ratio in *M. pontica* is at least three times greater than in *Pa. groenlandicus*. The lateral angle of the face of *M. pontica* is 5° greater than in *Pa.groenlandicus*, while the dorsal angle of the rostrum is 9° less (figures 1 and 13, and electronic supplementary material, table S7). *M. pontica* has a deep nasolabial fossa, which is shallower in *Pa. groenlandicus* (figure 13). The cranial edge of the palate of *M. pontica* reaches nearly half of the intraorbital region. The pm3, pm4 and m1 of *M. pontica* have only a main cusp, while in *Pa. groenlandicus*, mesial and distal accessory cusps are also developed. *M. pontica*’s mandible body reaches the maximal height at the level of the pm4, while the mandible body of *Pa. groenlandicus* reaches the maximal height at the level of the m1.

*M. pontica* has a low supraspinous fossa of the scapula (27% of the bone length at the level of the scapular spine) and *Pa. groenlandicus* has a higher supraspinous fossa of the scapula (40% of the scapula length).

The greater tubercle of *M. pontica* is higher than the head of the humerus. The humeral intertubercular groove is narrow (13% of the bone length), and the diaphysis in the head-to-medial-condyle edge has a straight shape. In *Pa. groenlandicus*, the greater tubercle is lower than the head. Its lesser tubercle is high and robust; the intertubercular groove is wide (20% of the bone length); and the diaphysis in the head-to-medial-condyle edge is curved (figure 4; electronic supplementary material, figure S6).

The lateral epicondyle of the femur of *M. pontica* is shorter than the medial one (the lateral epicondyle corresponds to 75% of the medial), which is relatively high and measures 50% of the total femoral height. In *M. pontica*, the greater trochanter of the femur is on the same level as the head. In comparison, *Pa. groenlandicus* has equal in size lateral and medial epicondyles, where the medial epicondyle reaches about 40% of the total femoral height. *Pa. groenlandicus* has a greater trochanter higher than the femoral head. The suprapatellar fossa of *Pa. groenlandicus* is more pronounced than that of *M. pontica* (figure 10).

#### *Histriophoca fasciata* (ZMUC CS-303-67)

*M. pontica* has an oval infraorbital foramen; on the contrary, *H. fasciata* has a rounded infraorbital foramen. Its DNFO/RH ratio is about three times greater than that of *H. fasciata* (figure 13). The lateral angle of *M. pontica*’s facial area (figure 1) is 5° less than that of *H. fasciata*, and the dorsal angle of the rostrum is 14° less (figure 13 and electronic supplementary material, table S7). *M. pontica* has a deep and narrow nasolabial fossa, which is shallow and wide in *H. fasciata*.

*M. pontica* mandible body reaches a maximal height at the level of pm4, while the mandible body of *H. fasciata* reaches a maximal height at the level of diastema between pm3 and pm4. *M. pontica* has a narrow intertubercular groove of the humerus (13% of the bone length versus 29% in *H. fasciata*); its trochlea is narrow (17% of the bone length versus 30% in *H. fasciata*) than the head, and its radial neck is 2.5 times thinner than the width of the distal part (versus 2 times thinner in *H. fasciata*) (figure 3). The femur of *M. pontica* has a medial epicondyle longer than the lateral one (130% of its length), while *H. fasciata* has the opposite (medial is 91% of the lateral one).

#### Phocini

#### *Phoca vitulina* (NMW 1462, 28587)

The DNFO/RH ratio of *M. pontica* is more than twice as great as that of *Ph. vitulina* (figure 13). Its lateral angle of the face is 10° less than in *Ph. vitulina* and the dorsal angle of the rostrum is 20° less than in *Ph. vitulina* (figures 1 and 13).

The smallest infraorbital width of *M. pontica* is 15% of the largest maxilla width, which is slightly thinner than 20% in *Ph. vitulina. M. pontica* has a deep nasolabial fossa. *M. pontica* has only the main cusp of the pm3, pm4 and m1. Its mandible body reaches a maximal height at the level of pm4. Whereas *Ph. vitulina* has shallow nasolabial fossa, well-developed main cusp, mesial accessory cusp, distal accessory cusp and sometimes cuspid, and the body of the mandible reaches a maximal height at the level of pm3.

*M. pontica* has a weakly developed supinator crest of the humerus [11] and the proximal bifurcation of the deltoid crest is absent (in *Ph. vitulina* this proximal bifurcation is present). The femoral greater trochanter is as wide as the diaphysis, and the medial epicondyle begins more proximal. While the trochanter of *Ph. vitulina* is wider than the smallest width of the diaphysis and the medial epicondyle is short (figure 10).

#### *Halichoerus grypus* (ZMUC 1485, NMW 28539)

The edge of *M. pontica* of the nasal cavity is concave in lateral view, While *Ha. grypus* has this edge straight. The DNFO/RH ratio is four-and-a-half times greater than in *Ha. grypus*, the lateral angle bordering the facial area is about ~25°, while *Ha. grypus* has the nasal bone almost parallel to the tooth row in the lateral view (figure 1). The dorsal angle of the rostrum is 14° less than in *Ha. grypus* (figure 13 and electronic supplementary material, able S7).

*M. pontica* has visible infraorbital foramen from the dorsal view. It has a deep and narrow nasolabial fossa. While *Ha. grypus* has hidden infraorbital foramen from the dorsal view (figure 13) and the nasolabial fossa is shallow and narrow.

*M. pontica* has only the main cusp of pm3, pm4 and m1, its mandible body reaches a maximal height at the level of pm4, and the chin prominence is pronounced. In contrast, *Ha. grypus* has a well-developed main cusp and the distal accessory cusp of pm3, pm4 and m1. Its mandible is equally high, and its chin prominence is slightly outlined.

The supraspinous fossa of the scapula of *M. pontica* is 27% of the bone length at the level of the scapular spine, the greater and lesser tubercles of the humerus are low but higher than the head, the proximal bifurcation of the deltoid crest is absent, and the anconeal crest of the humerus is weakly developed. While the supraspinous fossa of *Ha. grypus* is 30% of the bone length (figure 7), the greater and lesser tubercles of the humerus are high, the proximal bifurcation of the deltoid crest is present and the anconeal crest is well developed.

The head of the femur of *M. pontica* is faced in a medial–proximal direction, and the medial epicondyle is 130% of the length of the lateral epicondyle. While *Ha. grypus* has the head of the femur faced in a proximal direction and its medial epicondyle is 115% of the lateral one (figure 10).

#### *Pusa hispida* (ZMUC 803)

The DNFO/RH ratio in *M. pontica* is about twice that in *Pu. hispida*. The lateral angle of the face is 5° less and the dorsal angle of the rostrum is 17° less than in *Pu. hispida* (electronic supplementary material, able S7).

*M. pontica* has a deep nasolabial fossa, and its mandibular postcanine teeth have only the main cusp. In contrast, *Pu. hispida* has shallow nasolabial fossa, and its mandibular postcanine teeth have a strongly developed main cusp, mesial and distal accessory cusps, sometimes distal cuspid is also present.

*M. pontica* has a weakly developed supinator crest of the humerus, the proximal bifurcation of the deltoid crest bar of the humerus is absent; the medial epicondyle of the femur is 40% of the bone length and 130% of the lateral epicondyle. While the humeral supinator crest of *Pu. hispida* is strongly developed in the caudal direction, the proximal bifurcation of the humeral deltoid crest is present, and the medial epicondyle constitutes 35% of the bone length and 154% of the lateral epicondyle.

#### *Pusa caspica* (NMW 66292–66299, GNM 2-2013/988)

The DNFO/RH ratio in *M. pontica* is about 2.5 times greater than in *Pu. caspica*, the lateral angle of the face and the dorsal angle of the rostrum of *M. pontica* is 10° less than in *Pu. caspica* (figure 13; electronic supplementary material, able S7).

*M. pontica* has a deep nasolabial fossa, the maxillary tooth row is straight in lateral view, and the mandible body reaches the maximal height at the level of pm4. Only the main cusp is present. In contrast, *Pu. caspica* has deep and wide nasolabial fossa, the maxillary tooth row is slightly curved in lateral view. The maximal height of the mandible is at the level of m1, and *Pu. caspica* has a strongly developed main cusp, mesial and distal accessory cusps and distal cuspid.

In the humerus of *M. pontica*, the proximal bifurcation of the deltoid crest is absent, its anconeal crest is weakly developed, and the lesser tubercle is slightly higher than the head. *Pu. caspica* has the proximal bifurcation of the deltoid crest, the anconeal crest is strongly developed and the lesser tubercle of *Pu. caspica* is much higher than the head of the humerus (figure 3).

A greater trochanter of the femur is on the same level as the head and the same width as the diaphysis, the medial epicondyle of the femur is 130% of the lateral one, while the greater trochanter of *Pu. caspica* is higher than the head and wider than the smallest width of the diaphysis. The medial epicondyle is 115% of the lateral one (figure 10).

## 5. A review of other records of *M. pontica* (*Ph. pontica*)

### Seals from Gladkovskoye (Krasnodar Krai, Russia)

Podvincev *et al*. [74] briefly mentioned seal bones designated to *M. pontica* from Khersonian–Maeotian layers. The publication does not include any description or illustration, so its evaluation was impossible.

### Seals from Fortepianka (Adygea Republic, Russia)

According to Tarasenko [75], *M. pontica* bones were found in the top beds of the outcrop. Below in the sections, beds with *Chersonimactra caspia* are present. Considering the stratigraphic superposition of the fossiliferous beds, the beds with *M. pontica* could have Khersonian or Maeotian age. The authors identified seal bones as *M. pontica* [75]. Due to the lack of illustrations and collection numbers, the comparison and reassessment of the taxonomic attribution was impossible.

### *Seals from Kutsay Mountain*, *near Svetlograd (Stavropol Krai, Russia)*

Fossils found here are defined as ‘*M.* aff. *pontica* (slightly smaller than *M. pontica*)’ in Ivanov [76]. According to Ivanov [76], layers with *M. pontica* remains contain *Chersonimactra caspia* shells suggesting the Khersonian age. The publication does not include any illustrations and collection numbers of fossil findings which makes it impossible to reevaluate the identification.

### Seals from Küçükçekmece, Turkey

Material from Küçükçekmece includes two main elements:

1. A partial cranium (facial portion) described as *Ph. pontica* and illustrated by Malik & Nafiz [24]. Since the specimen was destroyed in a fire at Istanbul University in 1942, it was unavailable for reevaluation.
2. Specimens that are stored in the Muséum National d’Histoire Naturelle, Paris. The material includes mandible and postcranial material, which were redescribed by Peigné [77].

The comparison of the cranial material from Malik and Nafiz [24] is based on illustrations in Malik & Nafiz [24]. The rostrum has a DNFO/RH ratio of about 190%, which is very close to the same value in *M. pontica* (195%). The palatal process of the maxilla is slightly ventrally convex (this characteristic is shared with *M. pontica*). The upper and lower premolars of the Küçükçekmece specimen have the same character as *M. pontica*—well-developed main cusps of premolars. Thus, we suggest the specimen can be considered as *Monachopsis* sp.

Peigné [77] assigned humeral bones to *Cryptophoca* sp. However, some similarities between humeri MNHN.F.TRQ930, TRQ935 and humerus of *M. pontica* can be observed. Both humeri are similar in size to *M. pontica*. The humerus TRQ930 shares with *Cryptophoca* a diagnostic trait of the proximal part of the bone: the greater tubercle is lower than the head. The humerus TRQ935 has a long deltoid crest, which is slightly shorter than in *M. pontica* but longer than in *Cr. maeotica* and reaches the proximal edge of the epicondylar foramen. The femora MNHN.F.TRQ944 and TRQ945 belong to young individuals, and the smallest width of the diaphysis in the anterior view is slightly larger than half of the width of the distal epiphysis, which makes it closer to *M. pontica*. On the other hand, all bones are proportionally shorter than the bones of *Cr. maeotica.* In general, these bones have a transitional state in size and length of deltoid crest between *Cr. maeotica* and *M. pontica*. However, the material shows closer morphology to *M. pontica* and here is reidentified by us as a probable *Monachopsis* (?*Monachopsis* sp.).

### Seals from Dobrogea, Romania

Seals described by Chiriac & Grigorescu [54], as *Phoca pontica* from the Bessarabian deposits of Dobrogrea, Romania, slightly differ from *M. pontica* in having: more complex cusps of teeth, a shorter deltoid crest of humerus, more elongated radius, and an elongated wing of the ilium and about equal in size epicondyles of the femur*. M. pontica* has simpler teeth. It has only the main cusp while the Romanian specimens (LPB(FGGUB) 105 and 108) have additional tooth cusps. Most postcranial bones from Chiriac & Grigorescu [54] differ from *M. pontica.* A distal fragment of the humerus LPB(FGGUB) A6 has a shorter deltoid crest, which ends proximal to the proximal edge of the epicondylar foramen. Also, the femur LPB(FGGUB) A7 has a lateral epicondyle slightly shorter than the medial one. Its lateral epicondyle starts slightly distally than the middle of the femur. Whereas, the lateral epicondyle of the femur of *M. pontica* is twice shorter than the medial one and starts on the distal third of the femur. The specimen LPB(FGGUB) 157, which is mentioned in Grigorescu [10], differs from *M. pontica* in the more laterally situated distal part of the medial epicondyle, so this specimen is identified by us as *C.* cf. *maeotica*, distinguished for its small size. Thus, we conclude that published and illustrated findings from Romania refer not to *M. pontica* but to another Paratethyan seal.

## 6. Phylogeny

**Figure 14.**
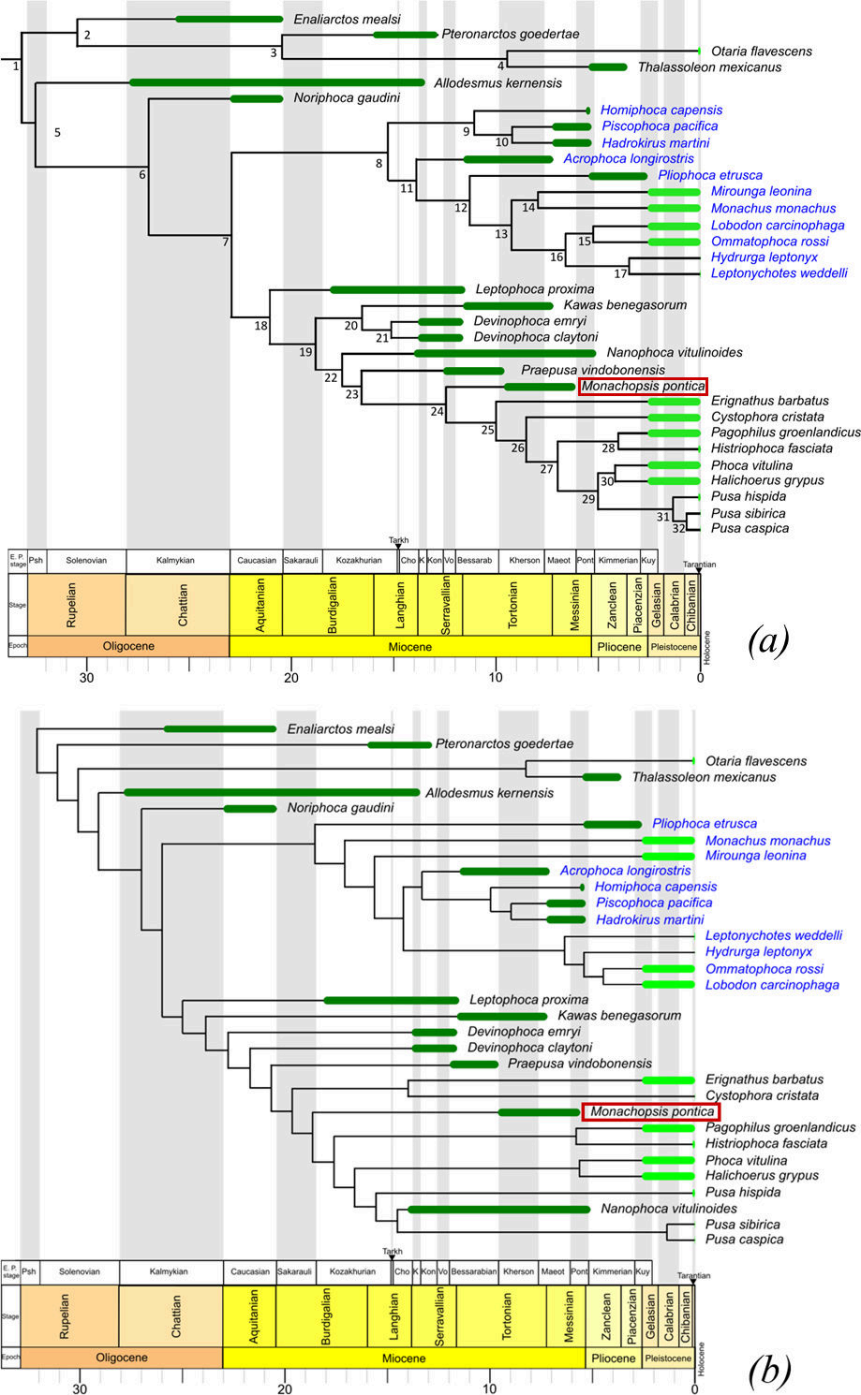
Phylogenetic analysis of true seals (Phocidae). (*a*) Total evidence tree. Nodes are numerated and for their posterior probabilities, age and 95% highest probability density, see electronic supplementary material, table S8. (*b*) Tree with implied weighting (*k* = 3), consistency index 0.356, retention index 0.709.

In the total evidence analysis, for most statistics, the effective sample size was more than 200 (except for mutation rates, which were more than 100). In this analysis, *M. pontica* forms the clade with all other modern Phocinae (figure 14*a*). The approximate diversification of *M. pontica* from other Phocinae was between 9.82 and 15.24 Ma (95% highest probability density; see electronic supplementary material, table S8). In the implied weighting tree, *M. pontica* forms a clade with *Nanophoca vitulinoides* and all crown phocines except *E. barbatus* and *C. cristata* (figure 14*b*).

## 7. Discussion

### 7.1. Probable adaptations to salinity changes

Phocidae is suggested to have originated in the Mediterranean region about 22 Ma ago [78,79] and colonized the Eastern Paratethys no later than in the Volhynian [46,80]. From the late Bessarabian, the Eastern Paratethys underwent several major water-level drops, each of which was followed by a transgression. In turn, the water-level dynamics caused water chemistry fluctuations affecting life in the marine realm. The terminal Bessarabian is characterised by a water-level drop, which killed almost the entire marine fauna [81–83]. During the following Khersonian substage, the Eastern Paratethys base level fluctuated constantly with the largest water-level drop during the second half of the Khersonian [14,84]. The water-level fluctuation was probably followed by changes in the salinity of the basin [15].

Due to these changes, the fauna of Paratethys was under strong ecological stress. Thus, many species of fish went extinct during the late Bessarabian–Khersonian. For example, recent studies made on findings from fish otoliths from Khersonian of Khroni Cape in the Kerch Peninsula show the complete extinction of stenohaline endemic fishes (Gaidropsaridae, Soleidae and some endemic groups of Gobiidae such as *Pontogobius*, and *Globogobius*) from Paratethys, and only euryhaline fishes remain (Clupeidae, Atherinidae, Sciaenidae, Gadidae, Gobiidae, Macroramphosidae, Bothidae and Serranidae) [9]. This extinction in the early Khersonian was called the ‘late Sarmatian s.l. extinction event’ [9,82]. In addition to the water-level fluctuations of the basin, the changes in water chemistry also could have played an additional role in the extinction process. Schwarzhans *et al*. [16] provide more pieces of evidence about the hypothesis that suboxic sea bottom could decrease benthic fish diversity in the Eastern Paratethys.

All these changes in the isolated ecosystem influenced the evolution and led to the divergence of Paratethyan marine mammals from their oceanic relatives. As a result, a high diversity of true seals was reached by the Bessarabian substage: at least four morphological groups (nominal genera: *Cryptophoca, Pachyphoca, Pontophoca, Praepusa*) of true seals have been described to date from the Bessarabian age of the Eastern Paratethys [11,18,22,64,66]. However, the Khersonian extinction events also impacted the diversity of true seals. So, *M. pontica* became one of the latest of them and survived in the Khersonian age. Another dated Khersonian seal record is *Praepusa procaspica*, described from the Eldari formation, Georgia [17]. *Pr. procaspica* was a small-sized form which was even smaller than *M. pontica*.

The environment where *M. pontica* lived was a marine coastal lagoon with a riverine input. These conditions could be favourable for *M. pontica* pachyostosis. According to [85], marine mammals that live in shallow water often have pachyostosis to control their buoyancy and easily reach the bottom of the sea where they often find their food resources. On the other hand, the pachyostosis in *M. pontica* could be inherited from ancestor species of Paratethyan seals [61]. They could live during the Badenian/Karaganian salinity crises followed by diversification [11]. Probably, the pachyostosis was useful for them to regulate buoyancy in marine water with increased salinity. The isolated Paratethyan basin protected Paratethyan seals from competition with new, more agile, open marine forms of seals and predators during the Bessarabian and Khersonian. However, pachyostosis increases the additional weight of the body which leads to a decrease in swimming speed and manoeuvrability.

As an adaptation to the massive skeleton, *M. pontica* developed an elongated infraspinous fossa of the scapula, a long deltoid crest of the humerus, and an enlarged medial epicondyle of the femur. The function of these structures was the attachment of swimming muscles. Namely the well-elongated infraspinous fossa and insertion of *m. infraspinatus* show that *m. infraspinatus* (used for increasing the power of abduction of forelimbs) was well developed. The extremely long deltoid crest also demonstrates the development of *m. atlantoscapularis*, *m. humerotrapezius* (used for the powerful extension of the forelimb), *m. deltoideus* (used in the powerful abduction of foreflippers) [86], and *m. pectoralis* (used for adduction of the foreflipper) [87]. So, *M. pontica* had comparatively powerful forelimbs, which could be important in both aquatic and land locomotion using them for paddling at low speeds or turning [88]. However, the large size (higher than the humeral head) of the lesser tubercle shows that *M. pontica* did not use forelimbs much for underwater propulsion, in contrast to ancestral Phocidae [86]. Meanwhile, hindlimbs should be actively used in propulsion: the enlarged medial epicondyle and medial part of the proximal part of the diaphysis reveal a well-developed *adductor magnus* muscle, which is used to adduct hind flipper during swimming [89]

### 7.2. Tooth wear and diet

The skull CH00-01 probably belongs to the ontogenetically older individual because it shows slightly pronounced sutures of the skull and strongly worn teeth. The wearing reaches teeth roots and crowns are completely absent. In modern Phocidae, strongly worn teeth occur only in *E. barbatus* and are connected with its glosswear suction feeding. During feeding, canines are worn by hard prey which leads to further wearing of postcanine teeth [90]. However, *M. pontica* does not show any other morphological specialization to suction feeding, like concavity on the palate or raised maxillary alveolar processes [91]. This may show that *M. pontica* was not specialized in suction prey capture and it fed by preferring the snapping method of prey capturing like most other true seals [92,93]. In addition, the tooth crowns of premolars are wider than narrow; however, unequal in size diastemata between them is in favour of an adaptation to raptorial feeding [94]. This way of feeding combines snapping and suction prey capture. As a result, animals with raptorial feeding can also use both methods of prey capture separately depending on prey size and type even without a specific morphological adaptation to suction [95].

Because the skull examined by us probably belongs to an ontogenetically old animal (see §3), its tooth wear could be caused by age. The animal could lose the ability to catch prey by piercing and start feeding by suction, which wears teeth furthermore. On the other hand, the skull NMNHU-P 64-516 also has a horizontally worn PM2 (while other teeth are absent), but its ontogenetic age is unclear. However, to prove if this wearing was an anomaly or a normal condition for older individuals, other material (skulls of young and adult animals, animals with transitional state of tooth wear) will be required.

### 7.3. Body size

*M. pontica* is one of the smallest seals from the Eastern Paratethys and one of the smallest known seals throughout their natural history The body size estimation suggests body length of adult individuals should be up to 105 cm. This places *M. pontica* among the smallest seals ever reported. According to our estimation, it had a similar size to *N. vitulinoides* for which estimated body length is 97.5 cm for the humerus IRSNB M2276c and 105 cm for IRSNB 1063-M242. This estimation is higher than the smallest previously estimated body size (51.7 and 55.9 cm) but smaller than the largest one (121.7 and 131.4 cm) [61]. Also, *M. pontica* is smaller than *A. changorum*, for which estimated body size is 128 cm, twice as large as the previous, original estimation of 68 cm [96]. *M. pontica* has similar sizes to ‘*Pr. boeska’* MAB 4686 (108 cm) and smaller than ‘*B. neerlandica’* MAB 3798 (88.5 cm) and the smallest taxa in our analysis *Pr. procaspica* (70 cm) (electronic supplementary material, table S4). Certainly, the absolute dimensions derived from allometric formulae will be revised, as new data are available; however, this broad comparison based on humerus size shows a diminutive size category for *M. pontica*.

Probably, the sea-level drop of the late Bessarabian and then a few times in Khersonian and, as a result, decreasing of the basin could lead to the decrease of the body size of *M. pontica* in comparison to its ancestors. Modern true seals show some intraspecies variation in body size due to phenotypic plasticity influenced by environmental conditions [52,97]. Some populations of *Pu. hispida,* which live in isolated basins, show a small average body size. For instance, *Pu. h. saimensis* average body size is 117 cm (up to 132 cm) [98] and *Pu. h. ladogensis* reaches up to 130 cm [99]. Also, the other smallest species of modern seals live in isolated basins. The Caspian seal *Pu. caspica* has an average body size of 130 cm and the Baikal seal *Pu. sibirica* has only 125 cm [100].

The absence of predators in isolated ecosystems could release predatory pressure on animals, so they do not need to outgrow predators or spend energy to escape them. As a result, the large animals may evolve into smaller forms [101,102]. Gigantic marine top predators disappeared in the Paratethys by the late Badenian, before the Volhynian. The latest records of *Otodus megalodon* came from the Badenian of Romania [103] and the Vienna basin [104]. Several findings of piscivorous Physeteroidea (but not killer sperm whales) also were reported from the Bessarabian and Khersonian substages of the Eastern Paratethys [105]. These species were unlikely to feed on *M. pontica*. As a result, during the Khersonian substage *M. pontica* had no predator pressure, so this could influence its body size decreasing.

### 7.4. Phylogenetic analysis

In both analyses, *M. pontica* is placed crownward to Miocene Phocidae, within the crown Phocinae and is most closely related to *E. barbatus* or *C. cristata* while other Paratethyan taxa belong to the stem part of the tree. However, its exact phylogenetic position within Phocidae remains unresolved, since in the total evidence analysis, it is placed at the base of the crown group inculding *E. barbatus*, while in the tree with implied weighting its place is crownward to *E. barbatus* and *C. cristata*. New specimens of Paratethyan seals and revision of nominal taxa are required to resolve the topology of *M. pontica.*

## 8. Conclusions

The newly described anatomical traits of *M. pontica* showed its uniqueness among the true seals and its relationship to living members of the subfamily Phocinae. The new description of dentition showed the raptorial feeding strategy of *M. pontica* with evidence of suction feeding. Phylogenetic analysis placed *M. pontica* at the base of the crown group of Phocinae. This gives us a clue to understand the relationship between modern and Miocene Paratethyan seals. The small body size, pachyosteosclerotic skeleton and body proportions of *M. pontica* can be explained by the absence of predators and the fluctuation of the water level in the fluctuating Paratethyan basin.

## Data Availability

All datasets supporting this article have been uploaded as electronic supplementary material [106].
